# Delay, deny, and defend: Public outrage at health insurance companies and stock market debacle

**DOI:** 10.1371/journal.pone.0334399

**Published:** 2025-10-14

**Authors:** Chen Li, Moumita Dutta, Jing Duo, Shantanu Dutta

**Affiliations:** 1 Telfer School of Management (Graduate Student), University of Ottawa, Ottawa, Canada; 2 Faculty of Medicine (Undergraduate Student), University of Ottawa, Ottawa, Canada; 3 Full Professor, Telfer School of Management, University of Ottawa, Ottawa, Canada; Yamanashi Gakuin University: Yamanashi Gakuin Daigaku, JAPAN

## Abstract

This study investigates the stock market’s response to the assassination of UnitedHealthcare’s CEO, focusing on the cumulative abnormal returns (CARs) of publicly listed U.S. insurance firms. Using topic modeling on 59,644 Reddit comments, we identify and analyze key public narratives surrounding the incident, revealing nine topics including the themes of (1) public anger at the profit-driven practices of the insurance industry, (2) support for the shooter, criticism of the CEO, and (3) frustrations over healthcare costs and systemic inefficiencies. Sentiment analysis further shows that discussions are overwhelmingly negative, reflecting widespread dissatisfaction. Empirical analysis demonstrates that corporate characteristics such as profit, revenue growth, and media attention significantly amplify negative CARs, highlighting the market’s sensitivity to perceptions of corporate profit and glamour. High executive compensation, particularly for CEOs, is also associated with more severe stock price declines, suggesting that leadership privileges intensify investor concerns. However, no evidence links CEO narcissism to negative stock impacts, indicating that public focus is more on systemic business practices than individual attitudes. Firms in the ‘Hospital & Medical Service Plans’ segment, including UnitedHealthcare and its peers, experienced the steepest declines immediately after the incident, reflecting heightened public discontent with companies closely tied to essential healthcare services. These findings support the views of ‘social banditry theory and investor sentiment’ and contribute to the broader debate between ‘shareholder vs. stakeholder value maximization’, emphasizing the risks of overlooking societal expectations.

## 1. Introduction

In the early morning of December 4th (Wednesday), CEO Brian Thompson of UnitedHealthcare – the largest U.S. insurer covering more than 50 million people – was assassinated in Manhattan, New York [1]. This incident subsequently led to a significant drop in stock prices of UnitedHealth Group, the parent organization of UnitedHealthcare [2]; the alleged killer, Luigi Mangione, was apprehended a few days after. The shooting has brought questionable business practices of the health insurance industry back into the limelight [3]. In parallel to UnitedHealth Groups’ [4] stock price debacle, the stock market also witnessed a ‘spill-over’ effect on other players in the insurance industry. After the shooting, bullet casings were found at the scene, inscribed with the words “deny, defend, depose” [5]. These words seem to imitate a common phrase used to describe central practices of insurance companies – “delay, deny, defend” – which are used to maximize profit and have been portrayed in media reports as disadvantaging policyholders seeking reimbursement [6]. These companies often delay the coverage process, deny the total or part of the original claim, or defend their decision through expert legal practitioners if they are disputed [7]. The murder has sparked renewed scrutiny on the practices described by the aforementioned phrase, as well as the insurance – and particularly the health insurance – industry.

Anecdotal evidence presented in news media outlets and social media platforms indicates that there has been an outburst of bitterness against insurance companies and their leaders, which leads to the sharp decline of insurance firms’ stock returns. The decline in stock performance of insurance companies seems to reflect the renewed sentiment of denouncing the aforementioned insurance practices by the general population, as well as the perceived apathy of the business leaders [3,6,8]. However, some insurance industry experts argue that extravagant profits among the insurance firms is a myth and hence negative public perception is misguided [9]. According to this view, there may not be any systematic relation between insurance company characteristics (such as past profitability) and stock return performance. In order to get a deeper insight into this issue, in this study, we empirically examine what causes the decline in stock returns among the publicly listed US insurance firms around the day of UnitedHealthcare CEO assassination. Using the ‘event study methodology’ we investigate the impact of the CEO shooting on stock returns across the insurance industry firms, and focus on the firm specific factors (such as profitability and glamour) and leaders’ compensation and personality traits that may explain the decline in stock returns. More specifically, we ask: do firm specific factors that are associated with opportunistic corporate profitability, glamour and corporate leadership traits (such as exorbitant CEO compensation and CEO narcissism) affect stock returns?

This question is motivated by the extant literature and news media coverage’s suggestion that the public outrage at insurance companies is primarily attributed to: ‘corporate profit and glamour’, and ‘corporate leaders’ privilege and personality traits’ – which could subsequently affect stock returns. These views are also in line with the ‘social banditry theory and investor sentiment’ and the perspective of ‘stakeholder theory’ (discussed in Section 2.5), which suggest that systems perceived as inequitable can unintentionally become a symbol of a community’s frustration that can manifest in various forms, eventually hurting the shareholder value of a firm due to narrow focus on the shareholder’s interests and corporate insiders such as CEOs – ignoring the welfare of other stakeholders (e.g., insurance clients). Accordingly, we focus on the above-mentioned factors and evaluate their effect on stock market performance of the U.S. insurance companies around the tragic event of UnitedHealthcare CEO assassination. For ‘corporate profit and glamour’, we use past profit, revenue growth, and media attention of insurance firms as relevant proxies. For ‘corporate leaders’ privilege and personality traits’ we use executive compensation and CEO narcissism as relevant proxies to examine the effect on stock returns.

Our empirical analysis demonstrates that corporate characteristics such as profit, revenue growth, and media attention significantly amplify negative CARs, highlighting the market’s sensitivity to perceptions of corporate opportunistic profit and glamour. High executive compensation, particularly for CEOs, is also associated with more severe stock price declines, suggesting that leadership privileges intensify investor concerns. However, no evidence links CEO narcissism to negative stock impacts, indicating that public focus is more on systemic business practices than individual attitudes.

We further document that firms receiving a higher volume of negative Reddit comments in the days following the event experienced significantly larger declines in stock prices. In a placebo test, we examine a sample of other sudden CEO deaths that did not trigger negative public discourse. We find no evidence of significant spillover effects to peer firms within the same industry. Moreover, in extended event windows beyond the initial three days, we observe that the cumulative effect on stock prices remains significant for most variables, with the magnitude of the coefficients initially decreasing but subsequently increasing, indicating a persistent adjustment process in investor reactions.

While sudden CEO deaths are often assumed to affect firm value negatively, prior studies suggest the overall mean stock market reaction to CEO death is often near zero, with large positive or negative reactions in individual cases depending on CEO and firm characteristics [10]. For example, the stock price reaction is significantly positive following the sudden death of entrenched executives [11]. Based on this literature, the assassination of UnitedHealth’s CEO would be expected to have only a minimal impact on the firm’s stock price, given that its CEO can be easily replaced at a low cost without materially affecting the firm’s operations [12]. This divergence from anticipated firm-level outcomes underscores the significant impact of public perceptions and investor sentiment on shaping market reactions during high-profile events.

Our study provides novel evidence that market responses to socially resonant events, such as sudden CEO deaths, can extend beyond firm-specific considerations and be shaped by public perceptions that influence investor sentiment across an entire industry. While we account for firm and CEO characteristics in our analysis, our main focus departs from prior work that interprets market responses to CEO deaths as reflections of the individual CEO’s impact on firm value [10–13]. In the context of the UnitedHealth CEO assassination, we show that the market response reflected broader public outrage and industry-wide negative investor sentiment. Furthermore, by applying social banditry theory, we provide new insights into the heterogeneity of these responses, revealing that companies perceived as exhibiting opportunistic profitability and executive privilege were punished by investors. Together, our findings carry broader implications for the shareholder–stakeholder debate. The stock price declines observed after the assassination challenge the core principle of the shareholder theory that corporate executives should focus solely on maximizing profits while disregarding social consequences, as UnitedHealth’s strong financial performance did not shield it from market backlash.

The paper proceeds as follows. Section 2 presents the background of the study and relevant hypotheses. More specifically, we discuss the stock market movement following the incident, overall challenges in the insurance industry, negative media sentiment following the assassination, the social banditry theory that explains negative sentiments following the event, and relevant hypotheses. Section 3 presents Reddit comments analysis – which reinforces the rationale behind our hypothesis; Section 4 presents the data, methodology, results for empirical analysis; and Section 5 presents the discussions.

## 2. Background and relevant hypothesis

### 2.1 The tragic incident and initial managerial acknowledgement

The tragic incident led to an intensive debate on the role of insurance firms and industry insiders, clients, and the general public started to reflect on the current practices of the insurance industry. Following the incident, CEO Andrew Witty of UnitedHealth Group – the parent organization that UnitedHealthcare is governed by – gave an originally private speech to the company saying that the company is a positive exemplar in the American healthcare system and will protect their business practices that ‘guard against unsafe or unnecessary care’ [5]. However, Witty outwardly acknowledges that many Americans frustrated with the perceivably inadequate healthcare system in place, and that there is definitely progress to be made. In his first public statement since the shooting, titled “The Health Care System Is Flawed. Let’s Fix It.”, he wrote that the healthcare insurance system is a ‘flawed patchwork built over decades’ and that it is ‘clear we have not reached a perfect system yet’ [6]. He later states that while they are partly responsible for some controversial coverage decisions, it is truly the purpose of their organization to ‘build a better healthcare system that works for everyone’ with the help of the government and community.

While there had been ongoing discussions on current practices in the insurance industry and political debate on how to restructure the policies and regulations, after a brief stable period, stock prices continued to decline for most insurance industry players even after two weeks of the unwarranted event of a CEO assassination. We focus on this aspect in the following sub-section.

### 2.2 Stock market movement following the CEO assassination

The stock market reactions to the tragic event of CEO assassination evolved quite strangely, as the market initially mainly focused on the recent disclosures and financial health of the company and largely ignored the implications of CEO killing and potential public outrage. The day that Brian Thompson passed away itself, it was a normal day for UnitedHealth Group Inc. in terms of their stock prices. The stock as almost completely flat, opening at $611.02 and closing at $610.79 [2]. This was a peculiarly stable performance, despite the assassination occurring a few hours prior to the stock market opening that morning; the stock value was even up by around a point for most of the day. It appears that the financial market was more focused on the financial aspects rather than the unfortunate event of an insurance giant’s CEO assassination. On December 3^rd^, UnitedHealth Group (NYSE: UNH) issued financial guidance ahead of its annual Investor Conference which was scheduled on December 4, beginning at 8:00 a.m. ET [14]. In that press release, the group projected 2025 sales of between $450 billion and $455 billion, which beats analysts’ consensus estimates of $431 billion. The financial market was content with UnitedHealth Groups’ future prospect and largely ignored the news of CEO assassination.

Meanwhile, the fatal shooting prompted a huge outpour of emotion in the general population [15]. Americans expressed their frustration, helplessness, and hostility towards insurance companies, some sharing personal anecdotes of their interactions with inscrutable mega-corporations. The incident unleashed a crashing wave of negativity not only onto UnitedHealthcare, but towards the insurance – and more specifically, the health insurance – industry as a whole. On social media, many posts criticized the strong profitability of health insurance companies in light of individuals’ reported challenges with coverage and reimbursement.

Due to this public outcry, UnitedHealth Group started experiencing difficulties the day after the murder [16]. Its share price declined sharply and continues to fall, losing more than $110 billion in market value by December 17^th^. The Dow Jones Industrial Average fell from 45,014.04 to 43,449.90 points during the same period, with a large portion (804 points) of the loss largely due to UnitedHealth Group and thus the assassination’s online reception. Furthermore, other health insurance companies are also experiencing similar losses; CVS Health Corporation decreased by 25%, Cigna Healthcare dropped by 20%, and Humana fell by 19%. All of these companies saw a monetary loss by the billions.

### 2.3 Problems in insurance industry

#### 2.3.1 A general lack of trust.

The public’s reaction following the shooting of UnitedHealthcare CEO Brian Thompson has all but underscored the general lack of trust towards health insurance companies; rather than an isolated act of violence, this incident is adjacent to growing frustrations over drawn-out dealings and rejected claims [17]. As much as there is sympathy for the deceased CEO being expressed on community forums and social media platforms, there is a much larger wave of criticism directed at UnitedHealthcare and health insurance companies as a whole. This incident has put a spotlight on the shift in public sentiment towards health insurance companies, moving from a detached apathy towards a focused animosity.

According to [17], the growing backlash against health insurance companies can be attributed to three “key failures” across all aspects of commercialism: not delivering value for customers, employees, and communities. First, customers feel that the high prices of goods and services – exacerbated the heavy strain of inflation – do not reflect an appropriate level of quality. Second, employees are feeling disillusioned over low wages, an unstable job market, and inflexible working conditions; the newer generations entering the workforce are particularly vocal about such issues. Finally, communities are not being properly served by self proclaimed ethical and principled companies; rather, they engage in “purpose washing” and conduct harmful practices under the radar.

Therefore, it is evident that the distaste towards health insurance companies is not a sudden phenomenon, but rather a culmination of gradual socio-economic frustrations; Systemic concerns across customers, employees, and communities, seem to have converged in their expression within this industry. Even the industry insiders admit the significant flaws: for example, after the shooting, UnitedHealthcare CEO Andrew Witty acknowledged that the US healthcare system is imperfect and that it must be improved upon through a collective effort [18].

#### 2.3.2 Tactics used by insurers - “delay, deny, and defend”.

One of the major criticisms of the health insurance industry is the use of the strategy “delay, deny, and defend”, which maximizes company profits by minimizing payouts. This term, coined in 2010 by Jay M. Feinman, has come to light in the general public’s eye as it was referenced on the bullet casings used in the killing of Brian Thompson. This connection to the current socioeconomic climate underscores the need to examine such practices, as negative public sentiment toward them has recently become more visible. Thus, it is particularly relevant to discuss the original meaning of this strategy and its implications on clients of insurance corporations.

First, ‘delay’ refers to companies prolonging the insurance claim process by establishing convoluted barriers (such as extensive forms) or simply taking a long time to respond in a debate regarding the claim. This is all done in the hopes that the claimants will settle for a lower amount or altogether abandon the claim in the first place. Second, ‘deny’ refers to offering a lower monetary amount for the claim or even outright rejection. This often occurs even if the grounds for the coverage amount are clearly valid. In this vein, insurers depend on the claimant’s lack of resources, time, and energy to pursue the matter further. Finally, ‘defend’ is a last resort, where companies will undertake prolonged legal battles; with their plethora of resources and highly skilled personnel, they have a high chance of overpowering the claimants and settling the coverage amount for a much lower amount than appropriate.

According to critics cited in media reports, the ‘delay, deny, defend’ approach may adversely affect patient recovery and survival [19]. UnitedHealthcare itself provides coverage to more than 50 million Americans; furthermore, as the shooting was directly targeted through their CEO, the corporation is thus facing increased scrutiny in the wave of criticism directed at health insurers in recent weeks [20].

### 2.4 Negative public sentiment: social media outcry and stock market reactions

In general, literature on news media and social media show that media content can sway investor sentiments and affect stock returns. Tetlock [21] find that negative news media coverage can robustly predict downward pressure on market prices. The finding suggests that media coverage serves as a proxy for investor sentiment. Tetlock et al. [22] posit that news media could contain certain soft information which is not captured by other publicly available information. Similarly, Dougal et al. [23] also find that financial journalists have the potential to influence investor behaviors.

A number of studies have also examined the relation between social media contents and stock market (e.g., [24–26]) and generally report that a higher proportion of negative social media posts/comments about a company leads to lower stock returns [27].

The assassination of UnitedHealthcare CEO on December 4^th^ led to a huge public outrage on social media platforms mainly criticizing the predatory practices and opportunistic behavior of insurance companies. Traditional news media also covered the story extensively and focused on the flawed practices in the insurance industry. Especially, the aftermath of Brian Thompson’s assassination saw an eruption of reactions across social media platforms, including TikTok, Instagram, X, Reddit, and Facebook; this was further concentrated and reported by news outlets and independent bloggers [8]. This in turn triggered a drop in UnitedHealthcare’s stock value.

For the first few hours after the incident and general reporting across news outlets, stock remained stable; however, the situation could not be more different the following days. The overarching corporation, UnitedHealth, lost 5.2% of its value, decreasing further as there was an outburst of vicious comments on social media, prompting the publication of multiple negative articles on UnitedHealthcare’s corporate practices [2]. Even as the company tried to salvage its image through executive announcements and appeals made with the help of their employees, it became evident that the damage was already done [28]. Social media saw a range of responses, from some users expressing sympathy for the CEO (primarily on professional networks such as LinkedIn), to others justifying the intent of the shooter [29,30]. Some posts were so vicious that X was compelled to remove them swiftly. In addition to such polarized perspectives, another significant portion of discourse surrounded users irately recounting their own experience with the healthcare insurance system.

Consequently, such negative sentiments shared through social media platforms and highlighted in traditional news media led to a meltdown of the insurance companies’ stock prices.

### 2.5 Relevant theories

#### 2.5.1 Social banditry theory and investor sentiment.

Under this theoretical framework, we attempt to explain why the public outrage towards insurance firms peaked after the shooting incident and how it eroded investor confidence that eventually led to a massive stock price decline.

While at first glance, the public reaction to the Brian Thompson’s assassination is alarming, some have pointed out that it aligns with long-standing sentiments held by the average citizen. ‘Social banditry’ theory, coined by Erix Hobsbawm, postulates that upholders of justice in an unjust society become widely admired and respected – particularly those that function antithetically to the law as well as against the rich and powerful [31]. This theory explains how individuals that combat inequitable systems can unintentionally serve a societal function by becoming a symbol of a community’s frustration; these ‘social bandits’ thus have the support and admiration of the working class, even if their actions are morally ambiguous [32].

The most well-loved exemplar of this theory is the legend of Robin Hood, who “robs the rich and gives to the poor” with dramatic flare [33]. A parallel can be drawn using the case at hand: a man leaving inscribed bullet casings – “deny, defend, depose” – parodying the industry after his crime [5], a wealthy company with a net worth of $500-plus billion, and a community that feels underserved by the law and healthcare. Unsurprisingly, ‘social banditry’ theory has been cited in reference to the shooting, with many framing the shooter as the embodiment of a ‘social bandit’ [34].

By targeting the executive of a company symbolizing opportunistic profit and social injustice, the meaning behind the shooter’s actions – whether intentional or not – resonates with a general populace that supported him through social media as a symbolic strike against the rich and powerful. Despite spending one-sixth of national GDP on healthcare, 31% of citizens are under or completely uninsured. For this reason, 25% of the population reportedly refrains from pursuing medical care and prescription drugs, to the detriment of their own well-being [34]. When faced with such statistics, it is evident why Americans, citizens of a developed nation, have grown increasingly jaded with the healthcare system and its adjacent insurance industry. Thus, the modern manifestation of social banditry in the shooting of Brian Thompson reflects the general public’s frustration and consequently a desire for justice against a perceivably inequitable system. As discussed in section 2.4, the general frustration with the insurance industry exploded following the shooting incident. Social media was full of vicious comments about health insurance criticism, unfair practices by insurance firms to patient advocacy, and frustration with insurance billing. Our analysis of Reddit comments (presented in Section 3) shows that most of the comments are negative in nature. Traditional news media also highlighted the flawed practices in the insurance industry and captured the negative perception of the general public towards the insurance industry.

From a financial perspective, this outburst of public outrage generated a powerful investor sentiment shock. Behavior finance literature has shown that negative news media and social media contents can affect investor sentiments and put a downward pressure on stock returns [21–24,26,27]. In line with this literature, the sentiment observed towards insurance companies reflects investors’ subjective valuations, possibly related to reputational or regulatory risks, rather than objective changes in firm fundamentals [35]. For example, negative discussions on social media may lead investors to fear that policymakers could introduce more stringent regulations, potentially affecting the future profitability of insurance companies. ‘Social banditry theory’ helps explain which firms were perceived as most culpable: those with the highest profits and greatest public glamour — typically viewed as success stories in financial terms — were seen by the public as the most exploitative and thus became focal points of outrage. Investors, anticipating that these firms would attract the most political scrutiny and become the primary targets of future regulation, reacted by aggressively selling their shares. This mechanism predicts that firms attracting stronger public backlash will experience deeper price declines.

Although no regulatory changes were enacted immediately, investors’ concerns were not unfounded. On December 11, 2024, lawmakers considered a bill aimed at breaking up healthcare conglomerates, which would have required large insurance firms to divest their pharmacy businesses. This development illustrates how public outrage can translate into real policy pressure. Although our study focuses on the immediate market response, UnitedHealth’s months-long price stagnation (till March end, 2025) highlights the sustained impact of sentiment-driven selling, triggered more by public backlash and investor overreaction than by changes in firm fundamentals. However, as earlier studies have pointed out, sentiment-driven sell-offs are often followed by long-term price reversals as investor expectations gradually realign with fundamentals [36]. Overall, ‘social banditry theory’, in combination with investor sentiment literature, provides a comprehensive framework for understanding both the direction and distribution of market reactions following the event.

#### 2.5.2 Shareholder vs. Stakeholder Theory.

The decline in insurance companies’ share prices can also be investigated by focusing on the relative importance placed on shareholder vs. stakeholder interests by the affected firms. What is the main goal of a business firm has been a central debate in the finance literature, often explored through the contrasting frameworks of shareholder and stakeholder theories. These perspectives have been instrumental in explaining a firm’s profitability, its impact on shareholders and other stakeholders, and its contribution to societal value creation [37,38].

Shareholder theory, originating from Friedman’s [39] proposition, asserts that the primary responsibility of a corporation is to maximize shareholder value. This perspective asserts that corporations are accountable solely to their shareholders, with no broader obligations to society beyond complying with applicable laws, ethical standards, and international norms [40]. According to this principle, corporate executives have a responsibility to conduct business in a way that makes as much money as possible [39].

In contrast, stakeholder theory, as proposed by Freeman [41], argues that corporations have broader responsibilities that extend beyond their shareholders to include all stakeholders impacted by their operations. Stakeholders encompass customers, employees, suppliers, communities, and society at large. This perspective emphasizes that firms must balance the interests of various stakeholders to achieve sustainable success and long-term value creation, challenging the profit-centric approach of shareholder theory.

Shareholder theory and stakeholder theory fundamentally differ on two key questions: to whom the corporate executives are responsible for and how social welfare is best improved. The shareholder theory asserts that executives are solely responsible for their shareholders, with the only responsibility of a business being to use its resources and engage in profit-generating activities [39]. Furthermore, it posits that social welfare is maximized when firms focus on profit generation, as this ensures that the value of outputs exceeds the costs of inputs in firms operation, thereby creating net value for society [42]. However, stakeholder theory posits that the interconnections between economic and social forces are complicated. As Freeman (41, p. 40) argues, ‘Isolating “social issues” as separate from the economic impact that they have, and conversely isolating economic issues as if they had no social effect, misses the mark both managerially and intellectually.’

In terms of shareholder theory, the performance of executives at UnitedHealthcare is impeccable. The company reported earnings exceeding $6 billion in the third quarter of 2024, with the revenue of its underlying business surpassing $100 billion [43]. However, the shooting on December 4^th^, 2024, exposed public frustration and challenges the notion that firm value maximization automatically translates into social welfare maximization. In fact, the widespread backlash on these firms after the shooting revealed a critical issue: their failure to deliver value to customers [17].

Customers, who in the case of insurance companies are typically policyholders, are key stakeholders in both business and financial terms. In the insurance context, policyholders also resemble debtholders, as their claims on the firm are fixed or contingent and take priority over shareholder claims in the event of insolvency [44,45]. This creates a dual role for policyholders: they are both the core clients whose satisfaction is central to the business model, and financial claimants whose protection is vital to the firm’s solvency and credibility. When management places excessive focus on profitability and firm valuation while neglecting the interests of policyholders, it can lead to public dissatisfaction and reputational harm. Given their dual role, policyholders naturally fall within the broader stakeholder group whose interests may conflict with those of shareholders. Our study examines stock price reactions and shows how perceived neglect of a critical stakeholder group can ultimately harm shareholder value.

While the contextual development of ‘social banditry theory and investor sentiment’ and ‘shareholder/stakeholder theory’ differs in nature, both have similar implications for the stock market reactions to insurance companies following the UnitedHealthcare CEO assassination. ‘Social banditry theory and investor sentiment’ captures people’s frustration due to perceived injustice to insurance firm clients which led to a negative reaction by investors; whereas the debate surrounding ‘shareholder vs. stakeholder theory’ highlights the potential consequences of ignoring the interests of broader stakeholders of a firm, which can eventually hurt the shareholder’s value itself.

### 2.6 Hypothesis development

As we have discussed in the earlier sections, following the assassination of the UnitedHealthcare CEO, insurance companies attracted negative publicity for profit-oriented business practices and insufficient attention to their clients’ needs. Post-assassination, social media platforms’ public outcry and conventional news media (e.g., newspapers) coverage analysis suggest that the public outrage at insurance companies is primarily attributed to ‘corporate profit and glamour’ and ‘corporate leaders’ privilege and personality traits’ – which could subsequently affect stock returns. The views are also in line with the ‘social banditry theory and investor sentiment’ – which implies that public outrage is directed more towards the riches and prominent entities and persons – and the ‘stakeholder theory’, which propagates that focusing narrowly on shareholder interests and corporate insider benefits could cost the shareholder value eventually.

Below, we discuss both aspects in more details, and present relevant hypotheses. We also performed a complementary social media analysis using Reddit comments (presented in Section 3), which reinforces the rationale behind our hypotheses.

#### 2.6.1. Corporate profit and glamour.

The insurance industry is often the target of attorney advertisements and external media, being painted as an imposing entity that is distrustful, callous, motivated to make opportunistic profit [9]. For example, one Georgia attorney promised to “pound money out of big insurance companies” in an advertisement for their services, representing these companies as piggy banks [46]. Furthermore, the Consumer Federation of America stated that “insurance company greed is the real inflationary pressure facing policyholders” [47]. According to a New York Police Department’s (NYPD) intelligence report, Brian Thompson’s assassination was likely driven by an overarching hatred for corporate greed manifested through exorbitant profits and the health insurance industry rather than a personal motive against the CEO [48]. A general perception is that insurance companies that make huge profit or experience a fast growth, do so at the expense of their clients’ wellbeing. Such public narratives contribute to negative investor sentiment, as investors anticipate reputational damage and regulatory backlash, which can lead to short-term overreactions and stock price declines, particularly for firms perceived as more exploitative.

**Hypothesis 1a:** Insurance firms with higher profit and higher revenue growth will have a more negative impact on the cumulative abnormal returns (CAR).

Earlier studies have argued that firms that attract more media attention are seen as ‘glamorous’ firms [49] - and such firms can attract more public anger around any negative corporate event. “Mass media are known to be powerful in directing the public’s attention towards specific issues, socially shaping individual’s opinions and investors’ behaviour in financial markets” [50]. In the case of the insurance company CEO assassination, intense media coverage amplified negative public sentiment toward the most visible and profitable firms. This public backlash, in turn, contributes to negative investor sentiment, as investors anticipate reputational harm or regulatory consequences, leading to sharper stock price declines for firms under heavier scrutiny. Accordingly, we present the following hypothesis.

**Hypothesis 1b:** Glamour firms with more media attention will have a more negative impact on the cumulative abnormal returns (CAR).

#### 2.6.2. Corporate leaders’ privilege and personality traits.

It is also evident that lots of public anger is directed towards CEOs of the insurance companies because of the perceived apathy and opportunistic behaviour of the insurance companies’ leaders [3,6,18]. Because of the online threats and derogatory remarks directed towards insurance company CEOs, many insurance companies feared for their executive teams and removed their photographs from company websites [51]. In this study, we focus on two CEO related aspects pertaining to their privilege and personality – which may drive public anger towards them: CEO compensation, and CEO narcissism.

General public and market participants care about the level of CEO pay; and an exorbitantly higher CEO pay draw their attention to the gulf between rich and poor [52]. Since the last few decades, public has started to care a lot about income inequality [53]. The fury over the high levels of pay for executives in the insurance industry was quite evident following the assassination a high-profile insurance company CEO. It did not go unnoticed that the UnitedHealthcare CEO, Mr. Thompson, earned a total compensation package last year of $10.2 million. An underlying perception is that the high paying CEOs of the insurance industry care only about the corporate profit and do not do enough to settle the claims of millions of their clients. For example, the division overseen by Mr. Thompson (CEO of UnitedHealthcare) reported $281 billion in revenue last year and provided coverage to more than 50 million Americans via plans for individuals, employers and people in government programs like Medicare and he received $10.2 million of compensation. However, many clients remained unsatisfied with the service of this insurance giant [54]. The disconnect between executive privilege and perceived client neglect not only fueled public anger but also contributed to negative investor sentiment, as market participants anticipated reputational damage and regulatory pressure for firms led by highly compensated CEOs. In light of this discussion, we present the following hypothesis.

**Hypothesis 2a:** Firms with higher CEO compensation will experience more negative effect on the cumulative abnormal returns (CAR).

Online discussions following the CEO assassination and precautionary measures undertaken by the insurance companies to project their executive suggests that public fury may also arise due to a CEO’s personality trait. One of the widely studied personality traits is CEO narcissism – however, there is no conclusive evidence in literature about the implications of this personality trait [55]. The American Psychiatric Association (APA, 2013) [56] describes narcissism as “a multifaceted personality trait that combines grandiosity, attention seeking, an unrealistically inflated self-view, a need for that self-view to be continuously reinforced through self-regulation, and a general lack of regard for others.”

Many experts have studied the effect of the level of CEO narcissism on corporate decision-making – however, conclusions often contradict. Some suggest that narcissistic CEOs are extraordinary visionaries and leaders, resulting in greater company organizational growth and innovation [57]; narcissistic CEOs also garner support by their charm and charisma [58,59]. On the contrary, others highlight that narcissistic CEOs are more likely to manifest a plethora of negative traits, including hubris, selfishness, and a propensity for unethical or illegal behaviour such as opportunistic insider trading [55,60]. The variety of findings on the relationship between CEO narcissism and corporate decision-making illustrates their interconnected intricacies and nuances.

In the context of this study, we take the view that narcissism will lead to ‘inflated ego’ among insurance company CEOs [55] which will result into lower apathy towards their clients. This perception can intensify public anger, particularly following the incident, as narcissistic CEOs may be viewed as embodying corporate arrogance and detachment. Such public reactions contribute to negative investor sentiment, as investors anticipate reputational fallout or heightened regulatory scrutiny for firms led by more narcissistic executives. This will lead to more public anger and affect stock returns negatively.

**Hypothesis 2b:** Firms with higher CEO narcissism will experience more negative effect on the cumulative abnormal returns (CAR).

#### 2.6.3 Additional analysis – impact on the targeted industry.

UnitedHealthcare is part of the ‘Hospital & medical service plans’ industry segment. Not surprisingly, insurance companies in this industrial segment attracted more public attention following the tragic event of the CEO assassination. Many of the social media posts are negative and discusses the unfair client treatments by the insurance companies in this segment. Similarly, the traditional news media coverage focuses on the overall problems persisted over the years in the insurance industry with a special focus on the companies belonging to ‘Hospital & medical service plans’ segment [6,54]. This surge in public and media scrutiny intensified negative public sentiment toward these firms, reinforcing their association with systemic injustice. Such public backlash can lead investors to anticipate reputational and regulatory risks, prompting short-term sell-offs and abnormal declines in stock prices.

**Hypothesis 3:** The effect of the shooting incident on the cumulative abnormal returns (CAR) is more severe on the insurance companies belonging to ‘Hospital & medical service plans’ industry segment.

## 3. Social media coverage: Reddit comments analysis

Our hypotheses are motivated by the visible public outrage towards insurance companies following the CEO shooting incident and the public perception on corporate profitability, glamour, CEO compensation, and personality trait of the insurance companies. To get a deeper insight into these potential mechanisms and to investigate public perception, we performed a complementary analysis using social media posts following the incident. More specifically, we used the Reddit API to collect relevant online comments from Reddit, a widely recognized platform for online discussions and public opinion analysis, and analyze those comments. In addition, we construct firm-level measures based on the volume of negative Reddit comments and examine their relationship with cumulative abnormal returns (CARs) in Section 4.8. These analyses provide direct textual evidence of the public’s emotional and critical responses, supporting our theoretical framework that links public outrage with investor sentiment and subsequent market reactions.

As one of the largest social media platforms, Reddit hosts diverse communities and facilitates open exchanges on various topics, making it a valuable resource for sentiment and discourse studies [61]. To achieve this goal, we employ the BERTopic model, a cutting-edge technique for topic modeling [62,63] to identify and categorize key themes emerging from the discourse. Additionally, we analyze how sentiments evolve surrounding the shooting incident involving the UnitedHealthcare CEO. This analysis aims to achieve two objectives: first, to examine the types of discussions and narratives that have emerged on Reddit in response to the shooting incident; second, to evaluate the extent and sentiment of the public’s reaction to this event.

### 3.1. Text data collection and BERTopic model implementation

We collected our comments data from Reddit (using Reddit API), comprising 69,726 original entries from posts with the query “Brian Thompson,” “Luigi Mangione,” and “UnitedHealthcare” between December 18 and 23, 2024. The posts we collected also cover three subreddit groups — “HealthInsurance,” “BrianThompsonMurder,” and “Luigi_Mangione” — capturing focused community discussions related to the incident. The data covers a substantial time range from December 4, 2024, to December 23, 2024, ensuring contemporary perspectives on the event. This broad scope provides a comprehensive basis for analyzing public opinion and thematic evolution over time.

Before performing the topic modeling analysis, the collected Reddit comments were subjected to a range of preprocessing techniques designed to improve the quality and suitability of the data for analysis [64]. These preprocessing methods were implemented to ensure the data was properly formatted for analysis and to enable BERTopic to accurately identify and interpret the semantic structures and meanings embedded in the text [63]. For data cleaning, the textual data was processed to remove emoticons, digits, punctuation, hyperlinks, redundant words, non-ASCII characters, and stopwords, as recommended by Xu et al. [65]. The Python scikit-learn library includes an English stopwords list, which comprises common words in English that carry minimal semantic or informational significance. These stopwords are usually removed in natural language processing tasks to improve the efficiency and precision of text analysis. Moreover, all uppercase letters in the dataset were converted to lowercase. To address potential redundancy due to overlapping data sources, duplicate texts were also filtered out. As a result, a total of 59,644 entries were retained for analysis.

We then apply the BERTopic model, which utilizes advanced language models to understand words within their specific contexts [64,66]. The capability of BERTopic allows it to identify intricate relationships among words, leading to enhanced accuracy in extracting topics from textual data. When applied to tasks like document clustering and topic assignment, BERTopic has shown greater effectiveness compared to traditional approaches [67]. Traditional topic models such as LDA and NMF often face challenges when applied to short and noisy texts, whereas BERTopic demonstrates superior performance in handling such data effectively [64]. Research has consistently shown that BERTopic surpasses LDA and NMF in terms of producing coherent and well-separated topics [64,67]. This suggests that BERTopic generates topics that are clearer and more logically structured. Additionally, BERTopic offers remarkable flexibility, making it suitable for analyzing diverse datasets, accommodating varying document lengths, and supporting multiple languages [68,69].

The BERTopic methodology, as outlined by Grootendorst [62], consists of three primary steps: generating numerical representations of documents through embedding, grouping documents into clusters, and applying the c-TF-IDF technique to analyze the clustered data. We use Sentence-BERT (SBERT) to transform preprocessed reddit comments into 384-dimensional embeddings, capturing semantic similarity [70]. To address the sensitivity of clustering algorithms like HDBSCAN to high-dimensional data [71], we apply Uniform Manifold Approximation and Projection (UMAP) for dimensionality reduction. UMAP effectively preserves the structural properties of data, similar to PCA and t-SNE [72]. When implementing HDBSCAN, we specified a minimum cluster size of 1,000 comments. As a result, 9 meaningful topics were identified, but 39,549 comments were classified as outliers. To reduce these outliers, we employed an approach based on cosine similarity, which was also utilized by Menezes et al. [63]. Using this method, all outliers were successfully reassigned to the 9 clusters.

### 3.2. Topic modeling

The shooting incident involving the health insurance CEO has sparked diverse discussions across social and political domains. Using BERTopic, we identified nine distinct topics that encapsulate the major themes of public discourse as shown in Table 1.

**Table 1:** Representative words and theme of each topic. This table presents the representative words and themes of the 9 topics clustered by the BERTopic model. Hypothesis relevance highlights how these topics provide support and context to our hypothesis development.

Topic	Representative words	Theme	Hypothesis relevance
1	health insurance, profit, cost, universal, industry, health care, universal healthcare, insurer, nonprofit, premium	Health insurance criticism	Hypothesis 1a
2	luigi, murder, thompson, shooter, mangiones, hero, prison, brian, support luigi, ceo, million	Support for Luigi and criticism for CEO	Hypothesis 2a, 2b
3	provider, visit, patient, hospital, appeal, billed, preventative, denial, clinic, screening	Patient advocacy and frustration with insurance billing	Hypothesis 3
4	medicaid, income, deductible, marketplace, tax, aca, employer, premium, enrollment, qualify	Healthcare system	Hypothesis 3
5	jury, trial, juror, charge, degree, murder, defense, attorney, judge, degree murder	Jury nullification and legal process debates	
6	backpack, mask, bus, bike, dna, police, camera, park, nypd, wearing	Details of the crime and investigation	
7	violence, revolution, protest, president, biden, trump, vote, violent, war, society	Political and social unrest	
8	speech, twitter, youtube, deleted, social medium, goodreads, free speech, screenshots, profile, channel	Free speech controversy and social media censorship	
9	lmao, gotcha, joke, thanks sharing, omg, ill look, youre welcome, he hot, bro, know thank	Social media interactions	

Public outrage toward the health insurance industry is directly targeted at its profit-seeking focus on maximizing shareholder returns. This sentiment is captured in Topic 1 which emphasizes systemic issues such as high “profit,” “cost,” and “premium,” alongside calls for “universal healthcare.” Many interpret the shooter’s actions as a response to the perceived exploitation within a model that prioritizes shareholder profits over patient care. For example, comments like “All the patients being denied healthcare for the sake of shareholder return are people too”,“Health Insurance profit is blood money”, and “Does scaring greedy CEOs count as terrorism?” highlight the public’s anger toward an industry widely criticized for valuing profits over individuals’ health and well-being. Discussions emphasize the inequities of the current system, which calls for reform that prioritizes “nonprofit” or government-supported solutions. This topic highlights the tension between corporate profit focus and public health, using the shooter’s actions as a lens to critique systemic failures. The public outrage discussed in Topic 1 is particularly relevant to Hypothesis 1a, which posits that insurance firms with higher profits and revenue growth will face more negative cumulative abnormal returns (CARs). The negative sentiment toward profit-driven practices, as evidenced in this topic, aligns with the hypothesis that firms perceived as prioritizing financial gains over client welfare are subject to harsher market reactions.

It may be argued that social media analysis (e.g., analysis of Reddit comments) alone is insufficient to capture the full spectrum of sentiment influencing the market. Indeed, this is why we extend our analysis beyond social media and include Hypothesis 1b, which is rooted in literature and focuses on the role of traditional media coverage in shaping stock market reactions. Previous studies suggest that firms attracting greater media attention are often perceived as ‘glamorous’ [49] and are more likely to draw public scrutiny and anger during negative corporate events.

Topic 2 captures public sentiment surrounding alleged shooter Luigi Mangione’s actions, integrating keywords such as “support Luigi,” “hero,” and “shooter,” which indicate that certain social media users expressed support for him, alongside terms like “CEO,” “million,” and “Thompson,” which reflect heated debates over corporate leadership. This support underscores widespread dissatisfaction with the healthcare system, with many perceiving it as prioritizing corporate profit over patient care. Comments like “Brian Thompson made 10.2mil a year through UHC including stocks, comps, salary etc.” and “Most C.E.O.s are narcissist. You can only climb to the top if you lack empathy” illustrate public frustration with high executive compensation and narcissistic leadership traits. The keywords in this topic, blending support for Luigi Mangione with criticism of CEOs and their earnings, directly connect to Hypothesis 2, which examines how executive characteristics, such as high compensation and narcissism, influence stock market reactions. This topic encapsulates the intersection of public support, anger at corporate leadership, and broader systemic frustrations with the healthcare industry.

Furthermore, a portion of the public has also focused on the patient group. In topic 3, keywords such as “provider,” “patient,” “hospital,” “billed” and “denial” suggest a focus on patient experiences and frustrations with healthcare services. The shooter’s actions have prompted many to share personal stories of denied claims, excessive medical bills, and inadequate access to care. This topic connects individual struggles with broader systemic issues, making the Luigi Mangione case a symbol of collective frustration. This topic is particularly relevant to Hypothesis 3, which posits that the shooting incident will have a more severe impact on the cumulative abnormal returns (CARs) of insurance companies in the ‘Hospital & Medical Service Plans’ industry segment. This segment includes firms such as UnitedHealthcare, Elevance Health, and their peers, which are the companies people typically turn to when dealing with medical expenses. Because of their direct role in managing healthcare payments and their visibility in patients’ financial and medical lives, these firms are uniquely positioned at the center of public dissatisfaction with healthcare costs and accessibility. As a result, companies in this segment are likely to experience the most significant market impacts, as they bear the brunt of the public’s anger and criticism following events like this one.

Similarly, the healthcare system itself comes under scrutiny in topic 4, as seen through words like “medicaid,” “income,” “deductible,” and “premium.” Public frustration with the complexity and financial burden of health insurance is evident. The shooter’s actions have further amplified calls for reform, with many tying their personal struggles with insurance companies to the broader systemic issues highlighted by the incident. This topic is also relevant to Hypothesis 3, which posits that the effect of the shooting incident on cumulative abnormal returns (CARs) will be more severe for firms in the ‘Hospital & Medical Service Plans’ industry segment. For industries tied to medical expenses, particularly those addressing regular healthcare needs such as doctor visits, hospital stays, and surgeries — the stock price declines are expected to be more pronounced, as these sectors face heightened public anger and criticism.

The Luigi Mangione case has also ignited broader discussions about societal and political tensions. Topic 5 explores the judicial proceedings surrounding the alleged shooter, focusing on terms like “jury,” “trial,” “attorney,” and “degree murder.” Many comments discuss jury nullification under this topic, suggesting it as a means to acquit the perpetrator despite legal guilt, reflecting widespread frustration with the healthcare system. For example, one commenter stated, “We need to spread the word about jury nullification - you can’t vote to find him not guilty,” while comparing Luigi’s actions to those against larger societal harms, emphasizing their anger toward healthcare companies. The legal debates emphasize the balance between justice for the crime and understanding the societal context of the shooter’s actions. Other topics, such as Topic 6, focus on the details of the crime. Keywords, such as “backpack,” “mask,” “bus,” and “dna”, point to discussions about the logistics and evidence of the crime. These details provide the foundational narrative for public discourse, focusing on how the crime was committed and the immediate response by law enforcement. In addition, in topic 7, terms like “violence,” “revolution,” “protest,” and references to leaders such as “biden” and “trump” indicate that the incident has become a flashpoint for broader frustrations with systemic inequality and government inaction. While some see the incident as symptomatic of deeper systemic problems, others use it as a platform for ideological debates, reflecting the polarized and contentious nature of modern political discourse. The public’s focus on terms like “revolution” and “protest” underscores the widespread desire for significant change.

In addition, Topic 8 focuses on debates about “free speech,” “twitter,” and “youtube,” reflecting discussions on platform policies, content moderation, and censorship. This topic reflects skepticism toward media platforms and disagreement with social media censorship. Topic 9 clusters informal and humorous commentary, including terms like ‘lmao,’ ‘joke,’ and ‘omg.’ These reactions provide limited substantive insight into the broader discussion of the incident, reflecting casual engagement rather than meaningful analysis.

### 3.3 Sentiment analysis on reddit comments

In this section, we use a fine-tuned DistilBERT model to analyze the sentiment of comments on the Reddit platform, aiming to assess the overall public sentiment toward the related topics. DistilBERT is a compact and efficient version of BERT, which is fine-tuned to classify English text into two categories: “Positive” and “Negative” with high accuracy [73]. This approach allows us to uncover the nuanced sentiments expressed in the posts while benefiting from the model’s speed and computational efficiency.

**Fig 1:**
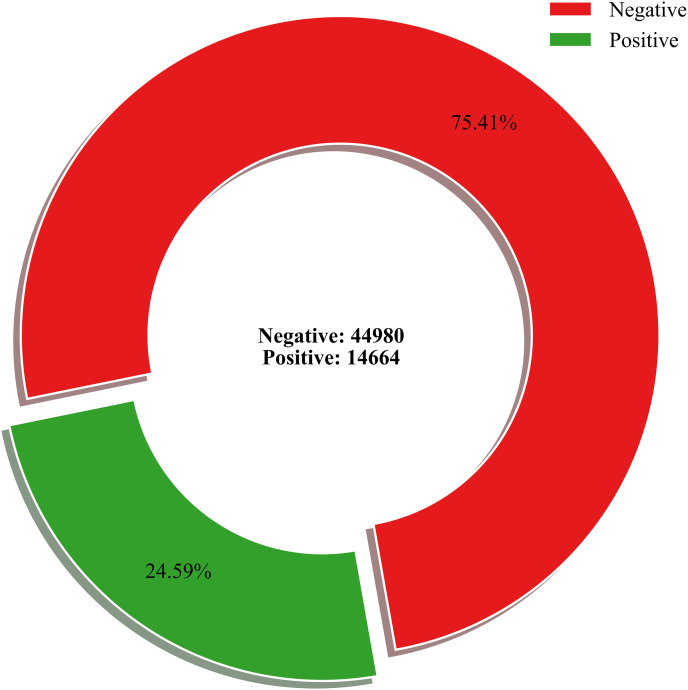
Sentiment distribution: A pie chart analysis.

Fig 1 shows the proportions of negative and positive sentiment in the Reddit comments sample. Of the 59,644 comments, 75.41% expressed negative sentiment and reflected significant public anger and dissatisfaction triggered by the event. Notably, 41.97% of the positive sentiment comments appeared in Topic 9 (Section 4.1.2), which captures casual internet interactions such as “LOL” or “Thank you.” Negative sentiment dominated all other topics and highlighted widespread public frustration with the insurance companies. The prevalence of negative sentiment throughout all topics suggests significant investor unease and potential adverse effects on the market perception of the insurance industry. To further investigate this, we test our hypotheses in the following sections using the cumulative abnormal returns (CARs) of insurance companies.

## 4. Empirical analysis and results

### 4.1. Stock sample, variable and methodology

#### 4.1.1.. Sample collection.

This study focuses on U.S. listed insurance companies’ stock performance surrounding the shooting incident that occurred on December 4th. We collect firms’ stock price data from the Compustat-CRSP Merged Database provided by Wharton Research Data Services (WRDS). We then identify insurance companies based on their Standard Industrial Classification (SIC) codes, as reported in their disseminated EDGAR filings which specify the type of their business. Following widely used Fama-French 48 category industry classification, we consider firms with SIC codes ranging from 6300 to 6411 as part of the insurance industry, yielding an initial sample of 150 firms. To calculate abnormal returns, we include only firms with stock price data available around December 4th, resulting in a final sample of 134 observations for our abnormal return analysis. We acknowledge that many studies focusing on insurance firms exclude the firms with the SIC 6411 code, which pertains to “Insurance Agents, Brokers & Service” [74,75]. However, our initial results indicate that firms in this category were also significantly affected by public sentiment (with a highly significant and negative abnormal return around this event; results are not reported here). Therefore, in the context of this study we have included this sub-category in our sample.

We collect executive compensation data from the Execucomp database available on WRDS. Since the database primarily includes S&P 1500 firms, we supplement missing values with executive compensation data extracted from DEF 14A filings using the SEC Filings API (https://sec-api.io/). For the market stock return, we obtain the indexes of S&P 500 from Yahoo finance. Besides the stock data, we obtain the firms’ accounting variables as our control variables from Compustat database. We then merge stock returns with firms that have available compensation and firm characteristic data, resulting in a final regression sample of 116 observations. We winsorize continuous variables at 1% level to address for outliers. Table 2 presents the summary statistics for the variables used in our analysis. Appendix 1 (supplementary information file: S2 Appendix) provides detailed definitions of these variables.

**Table 2:** Summary statistics. This table shows the sample descriptive statistics for the key variables used in the paper. There are 116 observations in the dataset. We report the sample size (N), mean, standard deviation (Std), 25th, 50th (Median) and 75th percentile statistics for the dataset. Detailed variable descriptions can be found in Appendix 1 (supplementary information file: S2 Appendix).

	(1)	(2)	(3)	(4)	(5)	(6)
Variables	Obs	Mean	Std. Dev.	25th Percentile	Median	75th Percentile
CAR	116	−0.017	0.034	−0.03	−0.016	0
Profit	116	−0.009	0.15	0	0.018	0.048
High_Rev_Growth	116	0.5	0.502	0	0.5	1
Media_1year	116	0.397	0.529	0.182	0.269	0.403
Media_3year	116	1.000	1.019	0.494	0.734	1.164
CEO_Compensation	115	8.527	1.123	7.909	8.774	9.268
Excu_Avg_Compensation	116	7.970	0.868	7.320	8.029	8.635
Excu_Total_Compensation	116	9.547	1.002	8.872	9.685	10.282
CEO_Narcissism	94	0.236	0.1	0.154	0.224	0.324
First_Pronoun	94	1.806	0.767	1.147	1.845	2.285
Size	116	8.996	2.293	7.384	8.878	10.83
Book_to_Market	116	0.761	0.964	0.351	0.735	1.076
Age	116	24.241	17.343	9	22	37.5
R&D	116	0.957	0.204	1	1	1
Leverage	116	0.118	0.127	0.031	0.072	0.153
Past_Return	116	0.504	0.862	0.169	0.349	0.562
Negative_comments	116	0.345	2.026	0	0	0
Negative_comments_dummy	116	0.052	0.222	0	0	0

#### 4.1.2. Main variable: cumulative abnormal returns.

We use the standard event-study methodology of [76] to analyze the impact of the shooting incident on the stock prices for the insurance firms. To analyze the stock market’s response, we construct Cumulative Abnormal Returns (CARs) as our main variable, which is a widely used measure of stock performance in event studies within the finance literature [77,78]. We compute the abnormal return around the incident day using a three-day event window [77]. Since the shooting incident is an accidental event and should not have any impact on the stock prices before the shooting date, we adopt an event window starting from exact the shooting date to two days after the shooting date. The abnormal return for firm *i* on day *t* is:


$$AR_{i,t}=R_{i,t}-E(R_{i,t})$$

where $AR_{i,t}$ represents the raw return of the common stock of firm i on day t, and $E(R_{i,t})$ is the expected return on stock i on day t. We use the market model to calculate the expected return on stock i:


$$E\left(R_{i,t}\right)=\alpha_{i}+\beta_{i}(R_{m,t})$$

where $R_{m,t}$ is the daily market return, and $\alpha_{i}$ and $\beta_{i}$ are calculated for each firm by regressing the firm’s returns on the market returns during the time period of 200 trading days to 30 trading days before the shooting date. Though the literature usually uses daily value weighted return from CRSP as market returns, but the data for 2024 is not available on CRSP when we conduct our analysis. Thus, we adopt the S&P 500 download from Yahoo Finance as market return proxy. The S&P 500 is a value-weighted portfolio of 500 of the largest U.S. stocks and is widely recognized as a benchmark proxy for the overall market [79]. Over the interval of three days around the shooting day, beginning with day 0 and ending with +2, the cumulative abnormal return, $CAR_{\text{0},\text{2}}$ (simply referred to as the CAR) is:


$$CAR_{\text{0},\text{2}}\;=\sum_{t=\text{0}}^{\text{2}}AR_{t}$$

Our first objective is to test if the CARs for insurance companies are significantly different from zero. We perform the following hypothesis test in Section 4.2:


$$H_{\text{0}}:\mu_{\text{1}}=\text{0}$$

where $\mu_{\text{1}}$ is the cumulative abnormal return. We use standard t-test statistics to determine the statistical significance of the results.

#### 4.1.3. Regression models.

To examine our hypothesis proposed in Section 2.6, we then use multiple regression models to examine the impact of firm and CEO characteristics on insurance companies’ CAR. For Hypothesis 1a and 1b, our regression models are defined as:


$$\begin{array}{c} CAR_{i}=a_{\text{0}}+\beta_{\text{1}}\;{Profit\;and\;Growth}_{i}+\beta_{\text{2}}{Size}_{i}+\beta_{\text{3}}{Book\_to\_Market}_{i}+\beta_{\text{4}}{Age}_{i}\\ \;+\beta_{\text{5}}{R\&D}_{i}+\beta_{\text{6}}{Leverage}_{i}+\beta_{\text{7}}{Past\_Return}_{i}+\epsilon_{i} \end{array}$$

$$\begin{array}{c} CAR_{i}=a_{\text{0}}+\beta_{\text{1}}\;{Glamour}_{i}+\beta_{\text{2}}{Size}_{i}+\beta_{\text{3}}{Book\_to\_Market}_{i}+\beta_{\text{4}}{Age}_{i}+\beta_{\text{5}}{R\&D}_{i}\\ \;+\beta_{\text{6}}{Leverage}_{i}+\beta_{\text{7}}{Past\_Return}_{i}+\epsilon_{i} \end{array}$$

where $CAR_{i}$ is the cumulative abnormal return for firm i within the three-day trading window around December 4th. ${Profit\;and\;Growth}_{i}$ is the explanatory variable for corporate profit and growth. Specifically, we employ two measures to capture this: Profit and High_Rev_Growth. $Profit_{i}$ measures the firm’s profitability, calculated as the ratio of Income Before Extraordinary Items (ib) to Total Assets (at). ${High\_Rev\_Growth}_{i}$ is a binary indicator that equals 1 if the firm’s average revenue growth rate over the past three years exceeds the sample median, and 0 otherwise. ${Glamour}_{i}\;$is measured by the total number of news articles mentioning the firm in the past one or three years in the Factiva database. We also control other firm characteristics in the model, including firm size, book-to-market ratio, and firm age. Appendix 1 (supplementary information file: S2 Appendix) provides detailed definitions of all variables used in the analysis.

We are primarily interested in the sign of $\beta_{\text{1}}$, which represents the impact of the explanatory variables on firms’ cumulative abnormal returns (CAR). Similarly, when testing alternative Hypothesis 2, we substitute the explanatory variables with executives’ compensation and their personal traits:


$$\begin{array}{c} CAR_{i}=a_{\text{0}}+\beta_{\text{1}}\;{Compensation}_{i}+\beta_{\text{2}}{Size}_{i}+\beta_{\text{3}}{Book_{to_{Market}}}_{i}+\beta_{\text{4}}{Age}_{i}+\beta_{\text{5}}{R\&D}_{i}\\ \;+\beta_{\text{6}}{Leverage}_{i}+\beta_{\text{7}}{Past\_Return}_{i}+\epsilon_{i}\; \end{array}$$

$$\begin{array}{c} CAR_{i}=a_{\text{0}}+\beta_{\text{1}}\;{Narcissim}_{i}+\beta_{\text{2}}{Size}_{i}+\beta_{\text{3}}{Book\_to\_Market}_{i}+\beta_{\text{4}}{Age}_{i}+\beta_{\text{5}}{R\&D}_{i}\\ \;+\beta_{\text{6}}{Leverage}_{i}+\beta_{\text{7}}{Past\_Return}_{i}+\epsilon_{i}\; \end{array}$$

where ${Compensation}_{i}$ represents the executive pay for firm i, we use three different proxies to measure the compensation level: the CEO’s individual compensation, the total compensation for all executives reported in the firm’s filings, and the average compensation level of executives. ${Narcissim}_{i}$ measures CEO narcissism and is calculated as the natural logarithm of one plus the ratio of first-person singular pronouns to all first-person pronouns used by the CEO during the Q&A section of earnings calls [80,81]. Finally, the regression model for testing Hypothesis 3 is:


$$\begin{array}{c} CAR_{i}=a_{\text{0}}+\beta_{\text{1}}\;{Hospital\&Medical\_Insurance}_{i}+\beta_{\text{2}}{Size}_{i}+\beta_{\text{3}}{Book\_to\_Market}_{i}\\ \;+\beta_{\text{4}}{Age}_{i}+\beta_{\text{5}}{R\&D}_{i}+\beta_{\text{6}}{Leverage}_{i}+\beta_{\text{7}}{Past\_Return}_{i}+\epsilon_{i}\; \end{array}$$

where ${Hospital\&Medical\_Insurance}_{i}$ is a dummy variable that equals 1 if the firm belongs to the same industry segment as UnitedHealthcare, identified by SIC code 6324. Since our sample comprises cross-sectional data with unique firms observed in the same year, we do not include firm or year-fixed effects. However, to enhance the robustness of our results, we incorporate additional control variables into the regression, despite the relatively small sample size. We also report robust t-statistics to ensure the reliability of our empirical findings.

One of the distinct advantages of our empirical set-up is that it is based on a truly exogenous event (CEO shooting) – this is a rare incidence in the North American corporate world, and no one was anticipating such an unfortunate event. This virtually eliminates the possibility of a ‘reverse causality’ in our empirical setup, which is a major concern in corporate finance empirical studies.

### 4.2. Economic effects of the shooting on insurance companies

**Fig 2:**
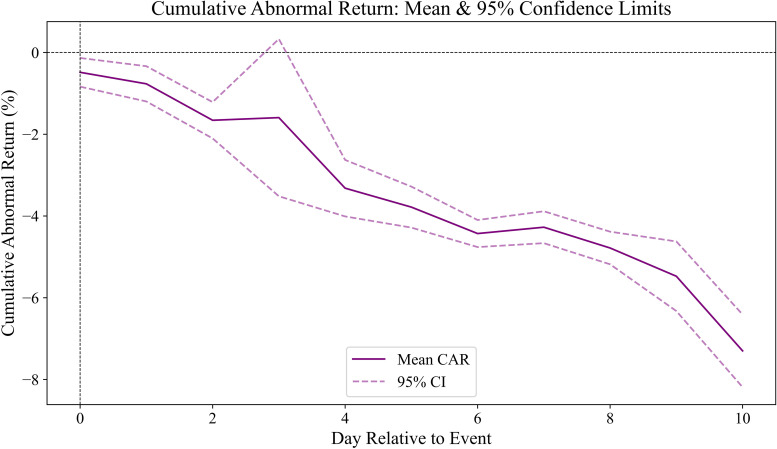
Cumulative abnormal return of insurance companies following the december 4th shooting incident.

Given the public anger and negative sentiment identified in Section 3, we examine whether the incident has a significant economic effect on the stock prices of insurance companies. To assess this impact, we analyze the cumulative abnormal returns (CARs) across various event windows, ranging from a three-day window (0, + 2) to an eleven-day window (0, + 10) following the shooting date [78]. Fig 2 illustrates the cumulative abnormal returns (CARs) for all insurance companies from ‘day 0’ (the shooting incident) to ‘day 10’. The CARs show a sharp decline in the first two days, reflecting an immediate negative market reaction. On day 3, a temporary rebound occurs, likely due to opportunistic investors attempting to buy the dip, given the substantial profit levels of these companies. However, this recovery is short-lived, as the negative sentiment and broader impacts of the shooting incident persist, causing CARs to continue declining over the subsequent days. The overall trend highlights the sustained adverse effects of the event on the insurance sector’s stock performance.

Table 3 presents the cumulative abnormal returns (CARs) for three sample sets. Panel A reports the CARs across various event windows for all insurance companies in the sample. The CARs for the six evaluated event windows are significantly negative at the 1% level, indicating a strong negative stock market reaction to the shooting incident among insurance firms. The CARs range from −1.658% (over the (0, + 2) window) to −7.299% (over the (0, + 10) window). Since news and reports about the incident began emerging on the second day, which could lead to public awareness later, we also examine windows starting from day + 1 (one day after the shooting), and the CARs remain significantly negative.

**Table 3:** Impact of December 4th shooting incident – univariate CAR analysis. This table presents the cumulative abnormal returns (CARs) for a number of windows surrounding the day of the shooting of the CEO of UnitedHealthcare on December 4th. Panel A presents CARs for all types of insurance firms, Panel B reports CARs for “hospital & medical service plans” insurance companies, while Panel C presents CARs for all other types of insurance companies. The classification of insurance companies are those with standard industry classification (SIC) code between 6300 and 6411. “Hospital & medical service plans” insurance companies, classified under SIC code 6324, provide prepaid or contract-based health services, including hospital, medical, and other healthcare services, to members or subscribers in exchange for specified subscription charges, with coverage often facilitated through prearranged agreements with healthcare providers. UnitedHealthcare and its competitors, including Elevance Health, Cigna Group, Humana Inc, and Centene Corp, all fall under this category. ***, **, * stand for statistical significance at the 1%, 5%, and 10% level, respectively.

Panel A. All Insurance Firms				
	# observations	Mean (%)	t-stat	p-value
CAR (0 to +2)	134	−1.658%	−5.424***	0.000
CAR (0 to +5)	134	−3.783%	−5.619***	0.000
CAR (0 to +10)	134	−7.299%	−6.745***	0.000
CAR (1 to +2)	134	−1.173%	−4.331***	0.000
CAR (1 to +5)	134	−3.298%	−3.622***	0.000
CAR (1 to +10)	134	−6.814%	−6.122***	0.000
**Panel B. Hospital & Medical Service Plans**				
	# observations	Mean (%)	t-stat	p-value
CAR (0 to +2)	10	−4.216%	−2.671***	0.008
CAR (0 to +5)	10	−6.875%	−4.731***	0.000
CAR (0 to +10)	10	−11.158%	−4.418***	0.000
CAR (1 to +2)	10	−3.928%	−2.700***	0.007
CAR (1 to +5)	10	−6.587%	−4.605***	0.000
CAR (1 to +10)	10	−10.870%	−4.243***	0.000
**Panel C. All Other Insurance Firms**				
	# observations	Mean (%)	t-stat	p-value
CAR (0 to +2)	124	−1.452%	−4.843***	0.000
CAR (0 to +5)	124	−3.534%	−4.946***	0.000
CAR (0 to +10)	124	−6.988%	−6.751***	0.000
CAR (1 to +2)	124	−0.951%	−3.651***	0.000
CAR (1 to +5)	124	−3.033%	−4.356***	0.000
CAR (1 to +10)	124	−6.487%	−6.383***	0.000

We further analyze insurance firms that have the closest relationship with UnitedHealthcare by focusing on those within the same industry, identified by sharing the same SIC code. Panel B of Table 3 reports the CARs for insurance companies with a SIC code of 6324, which includes UnitedHealth and many of its competitors. We find all CARs are significantly negative in this industry, the CARs range from −4.216% (over the (0, + 2) window) to −11.158% (over the (0, + 10) window). Panel C of Table 3 reports the CARs for all other insurance firms not included in Panel B. While the CARs are quantitatively smaller, they remain significantly negative.

Overall, the shooting incident represents a significant economic event, leading to substantial stock price declines for all insurance firms, with particularly severe impacts on the category that includes UnitedHealth and its competitors.

### 4.3. Corporate profit and cumulative abnormal returns following the shooting

From this section, we apply the multiple regression model introduced in Section 4.1.3 to examine which firm characteristics contribute to more negative CARs among insurance firms. Our analysis begins with testing Hypothesis 1a. As we found in Section 4.1, the public expressed significant anger and negativity on online platforms toward insurance companies, specifically criticizing their overemphasis on profit and revenue growth, which has generated widespread dissatisfaction. For example, we observed comments such as, “You don’t have to be insured to know how greedy the insurance companies have been,” and “No one should be profiting millions from healthcare, healthcare services, and medications… all this is critical to all Americans.” If customers and investors share these concerns, we would expect firms with higher profits and revenue growth compared to their peers to experience more negative CARs, leading to a negative coefficient for the explanatory variable.

We begin by regressing CARs on the firms’ profit and high revenue growth to test the hypothesis that higher profits and revenue growth lead to more negative abnormal returns. Table 4 presents the regression results across four specifications. The coefficient on Profit is negative and statistically significant at the 5% level in columns [1] and [3], with values of −0.045 and −0.047, respectively. This indicates that higher profitability is associated with more negative CARs, supporting the hypothesis that public and investor dissatisfaction with profit-driven motives impacts stock performance negatively. The coefficient on High_Rev_Growth is consistently negative and statistically significant at the 1% level in column [2] (−0.018) and at the 5% level in column [4] (−0.017). This finding suggests that firms with higher revenue growth experience greater stock price declines, potentially reflecting public frustration with aggressive growth strategies.

**Table 4:** Relationship between corporate profit, glamour, and cumulative abnormal returns (CARs) following the fatal shooting of the CEO. This table presents the relationship between the firms’ profit and revenue growth with the cumulative abnormal returns (CARs) after the day of the shooting of the CEO of UnitedHealthcare. The dependent variable is the cumulative abnormal return (CAR) from day 0 to day 2. In column (1) and column (3), CAR is regressed on firm profit, while in column (2) and column (4), CAR is regressed on a dummy variable indicating high revenue growth. The variable High_Rev_Growth is a binary indicator that equals 1 if the firm’s average revenue growth rate over the past three years exceeds the sample median, and 0 otherwise. Several firm accounting and stock characteristics are controlled for in the regressions, including firm size, book-to-market ratio, and firm age. Definitions of variables are provided in Appendix 1 (supplementary information file: S2 Appendix). Robust t-statistics are in parentheses. Asterisks *, ** and *** indicate the significance levels of 10%, 5% and 1%, respectively.

	(1)	(2)	(3)	(4)
Variables	CAR	CAR	CAR	CAR
Profit	−0.045**		−0.047**	
	**(−2.091)**		**(−2.041)**	
High_Rev_Growth		−0.018***		−0.017**
		**(−2.942)**		**(−2.547)**
Size	−0.000	−0.002	−0.001	−0.002
	(−0.226)	(−1.093)	(−0.385)	(−1.038)
Book_to_Market	−0.004	−0.006	−0.005	−0.007
	(−0.842)	(−1.398)	(−0.986)	(−1.510)
Age	0.000	−0.000	0.000	−0.000
	(0.556)	(−0.171)	(0.354)	(−0.316)
R&D			0.026*	0.023
			(1.762)	(1.587)
Leverage			−0.032	−0.026
			(−1.056)	(−0.780)
Past_Return			−0.002	−0.000
			(−0.444)	(−0.000)
Constant	−0.013	0.014	−0.028	−0.003
	(−0.806)	(0.914)	(−1.087)	(−0.116)
Observations	116	116	116	116
R-squared	0.058	0.098	0.105	0.131

The economic significance of the regression results is particularly large. A one standard deviation increases in Profit or High_Rev_Growth, the CARs of insurance companies decrease by 37.9% or decreases by 53.2% relative to the sample average CAR, respectively (The standard deviation of Profit and High_Rev_Growth is 0.15 and 0.502. For regressions in columns (1) and (2), coefficients of -0.045 and -0.018 reflect changes in CARs of -39.7% ( −0.045*0.150/0.017) and -53.2% ( −0.018×0.502÷0.017), respectively. As reported in Table 1, CAR has a mean value of −0.017).

We further divide the sample based on whether firms have positive or negative profitability, motivated by the observation in Table 2 that the median profit is positive and the possibility that the market may interpret profit levels differently depending on firms’ financial condition. The results show that the coefficient on profit is significantly negative only among firms with positive profitability, while it is statistically insignificant among loss-making firms. This suggests that public anger and investor backlash was concentrated on firms that were profitable before the incident, reinforcing the interpretation that visible profit-taking prior to the event became a focal point of social criticism and market reaction.

The results provide strong evidence that, following the shooting, higher profitability and revenue growth in insurance firms significantly amplify negative stock price reactions. This finding aligns with the hypothesis that public and investor dissatisfaction with profit-driven behaviors is reflected in market outcomes.

### 4.4. Media attention and cumulative abnormal returns following the shooting

In this section, we examine Hypothesis 1b, which posits that insurance firms with greater media attention experience more negative impacts on their CARs. To test this, we replace the explanatory variable in the multivariable regression model with the measure based on the number of media reports over the past one and three years, respectively. This approach follows prior studies that use media coverage frequency as a proxy for firms’ media exposure [82,83]. If the negative sentiment observed in Section 4 is reflected in the CARs, we would expect a negative coefficient for media coverage.

Table 5 reports the results. In columns [1] and [3], the independent variable is media attention over the past one year, measured as the number of articles related to the firm in the Factiva database. The coefficient for media attention is negative and statistically significant at the 1% level in column [1] (−0.020) and at the 1% level in column [3] (−0.019), suggesting that **f**irms with higher media attention in the past year experience more negative CARs. In columns [2] and [4], the independent variable is media attention over the past three years, measured in the same way. The coefficient is also negative, with statistical significance at the 10% level in both column [2] (−0.010) and column [4] (−0.009). This indicates that extended media attention over a longer period is similarly associated with more negative stock price reactions.

**Table 5:** Relationship between media attention and cumulative abnormal returns (CARs) following the fatal shooting of the CEO. This table presents the relationship between the median attention with the cumulative abnormal returns (CARs) after the day of the shooting of the CEO of UnitedHealthcare. The dependent variable is the cumulative abnormal return (CAR) from day 0 to day 2. In column (1) and column (3), CAR is regressed on the median attention in the past one year, while in column (2) and column (4), CAR is regressed on the median attention in the past three years. The median attention is measured as the number of articles (in thousands) related to the firm in the Factiva database in the past one or three years, respectively. Several firm accounting and stock characteristics are controlled for in the regressions, including firm size, book-to-market ratio, and firm age. Definitions of variables are provided in Appendix 1 (supplementary information file: S2 Appendix). Robust t-statistics are in parentheses. Asterisks *, ** and *** indicate the significance levels of 10%, 5% and 1%, respectively.

	(1)	(2)	(3)	(4)
Variables	CAR	CAR	CAR	CAR
Media_1year	−0.020***		−0.019***	
	**(−7.494)**		**(−4.832)**	
Media_3year		−0.010***		−0.009***
		**(−4.971)**		**(−3.448)**
Size	0.000	0.001	−0.000	0.000
	(0.188)	(0.416)	(−0.147)	(0.012)
Book_to_Market	−0.006	−0.006	−0.007	−0.006
	(−1.222)	(−1.160)	(−1.428)	(−1.341)
Age	0.000	0.000	0.000	0.000
	(0.232)	(0.177)	(0.075)	(0.014)
R&D			0.003	0.008
			(0.216)	(0.534)
Leverage			−0.037	−0.036
			(−1.244)	(−1.170)
Past_Return			−0.001	−0.001
			(−0.188)	(−0.165)
Constant	−0.008	−0.010	0.000	−0.006
	(−0.500)	(−0.595)	(0.005)	(−0.239)
Observations	116	116	116	116
R-squared	0.114	0.099	0.132	0.119

Overall, the results support Hypothesis 1b, which posits that glamour firms with greater media attention experience a more negative impact on cumulative abnormal returns (CARs). The significant negative coefficients for media attention in both the one-year and three-year periods indicate that firms with higher public visibility and scrutiny face stronger adverse stock price reactions following the incident.

### 4.5. Executive compensation and cumulative abnormal returns following the shooting

In this section, we test Hypothesis 2a, which posits that firms with higher CEO compensation will experience a more negative impact on cumulative abnormal returns (CARs). To evaluate this hypothesis, we include three executive compensation measures, all transformed using their natural logarithms, as explanatory variables in the multiple regression model to analyze their relationship with CARs following the incident. These measures are [1] the logarithm of CEO compensation, [2] the logarithm of the total compensation of all executives reported by the firm, and [3] the logarithm of the average compensation of executives within the firm. Together, these measures capture both the compensation of the top executive (CEO) and the overall level of executive compensation observed by investors, providing a comprehensive and scaled view of how executive pay influences market reactions.

Table 6 reports the results. Columns [1] and [4] show that CEO compensation is significantly negatively associated with CARs, with coefficients of −0.015 and −0.013, respectively, both significant at the 5% level. This suggests that firms with higher CEO pay experienced more negative stock price reactions. Columns [2] and [5] examine the average compensation of executives. The results indicate a negative relationship with CARs, with coefficients of −0.022 (significant at 1%) and −0.021 (significant at 5%), highlighting that firms with higher average executive compensation faced stronger negative abnormal returns. Columns [3] and [6] analyze the total executive compensation. The coefficients, −0.019 and −0.018, are both significant at the 5% level, providing further evidence that higher total executive compensation exacerbates negative stock price reactions.

**Table 6:** Relationship between Executive Compensation and Cumulative Abnormal Returns (CARs) Following the Fatal Shooting of the CEO. This table presents the relationship between the executive compensation with the cumulative abnormal returns (CARs) after the day of the shooting of the CEO of UnitedHealthcare. The dependent variable is the cumulative abnormal return (CAR) from day 0 to day 2. In column (1) and column (4), the dependent variable CAR is regressed on the natural logarithm of the CEO’s total compensation. In column (2) and column (5), CAR is regressed on the natural logarithm of the average compensation of all executives reported by the firm. In column (3) and column (6), the independent variable is the natural logarithm of the total compensation value of all executives reported by the firm. Several firm accounting and stock characteristics are controlled for in the regressions, including firm size, book-to-market ratio, and firm age. Definitions of variables are provided in Appendix 1 (supplementary information file: S2 Appendix). Robust t-statistics are in parentheses. Asterisks *, ** and *** indicate the significance levels of 10%, 5% and 1%, respectively.

	(1)	(2)	(3)	(4)	(5)	(6)
Variables	CAR	CAR	CAR	CAR	CAR	CAR
CEO_Compensation	−0.015**			−0.013**		
	**(−2.453)**			**(−2.132)**		
Excu_Avg_Compensation		−0.022***			−0.021**	
		**(−2.630)**			**(−2.184)**	
Excu_Total_Compensation			−0.019**			−0.018**
			**(−2.362)**			**(−2.007)**
Size	0.003	0.005	0.006	0.002	0.005	0.005
	(1.136)	(1.603)	(1.613)	(0.700)	(1.237)	(1.226)
Book_to_Market	−0.005	−0.008	−0.009*	−0.006	−0.008	−0.009*
	(−1.029)	(−1.638)	(−1.757)	(−1.139)	(−1.605)	(−1.770)
Age	0.000	−0.000	−0.000	−0.000	−0.000	−0.000
	(0.125)	(−0.431)	(−0.379)	(−0.051)	(−0.492)	(−0.488)
R&D				0.024*	0.024*	0.024*
				(1.763)	(1.749)	(1.660)
Leverage				−0.032	−0.007	−0.016
				(−1.192)	(−0.220)	(−0.527)
Past_Return				−0.001	0.001	0.000
				(−0.198)	(0.322)	(0.082)
Constant	0.086**	0.119***	0.125**	0.067*	0.092*	0.098*
	(2.557)	(2.688)	(2.329)	(1.839)	(1.933)	(1.723)
Observations	115	116	116	115	116	116
R-squared	0.151	0.143	0.126	0.199	0.168	0.155

Overall, the results support Hypothesis 2a, indicating that firms with higher CEO and executive compensation are more negatively impacted in terms of CARs following the incident. These findings suggest that public and investor scrutiny of executive pay plays a critical role in shaping market reactions to such events.

### 4.6. CEO narcissism and Cumulative Abnormal Returns following the shooting

In this section, we test Hypothesis 2b, which posits that firms with higher CEO narcissism will experience more negative effects on cumulative abnormal returns (CARs). To test Hypothesis 2b, we construct a measure of CEO narcissism measured as the natural logarithm of one plus the ratio of first-person singular pronouns to all first-person pronouns used by the CEO during the Q&A section of earnings calls [80,81]. This measure is incorporated as an explanatory variable in our multivariable regression model, alongside controls for firm characteristics, including size, age, and book-to-market ratio.

Table 7 presents the results of regressions analyzing the relationship between CEO narcissism and cumulative abnormal returns (CARs) for insurance firms following the shooting of UnitedHealthcare’s CEO. In column [1], CEO narcissism is measured as the natural logarithm of one plus the ratio of first-person singular pronouns to all first-person pronouns used by the CEO during the Q&A section of earnings calls. The coefficient for this variable is positive (0.026) but not statistically significant, indicating no strong evidence that this measure of CEO narcissism significantly influences CARs. Column [2] uses the original ratio of first-person singular pronouns as the independent variable. The coefficient (0.004) is again positive but lacks statistical significance, suggesting that this alternative measure of narcissism also does not have a meaningful impact on CARs. Similarly, we do not find significant results on CEO narcissism or first-person pronouns in columns [3] and [4]. As a robustness test, we use an alternative measure of CEO narcissism – calculated as the ratio between the total compensation of the CEO and that of the next-highest-paid executive in the firm [73]. This measure also leads to insignificant results.

**Table 7:** Relationship between CEO Narcissism and Cumulative Abnormal Returns (CARs) Following the Fatal Shooting of the CEO. This table presents the relationship between the CEO Narcissism and the cumulative abnormal returns (CARs) after the day of the shooting of the CEO of UnitedHealthcare. In columns (1) and (3), the independent variable is CEO narcissism, measured as the natural logarithm of one plus the ratio of first-person singular pronouns to all first-person pronouns used by the CEO during the Q&A section of earnings calls. In column (2) and (4), the independent variable is the original ratio of the first-person singular pronouns used by the CEO during the earnings calls. Several firm accounting and stock characteristics are controlled for in the regressions, including firm size, book-to-market ratio, and firm age. Definitions of variables are provided in Appendix 1 (supplementary information file: S2 Appendix). Robust t-statistics are in parentheses. Asterisks *, ** and *** indicate the significance levels of 10%, 5% and 1%, respectively.

	(1)	(2)	(3)	(4)
Variables	CAR	CAR	CAR	CAR
CEO_Narcissism	0.026		0.019	
	(0.671)		(0.527)	
First_Pronoun		0.004		0.003
		(1.129)		(0.761)
Size	−0.008	−0.010	−0.025	−0.025
	−0.002	−0.002	−0.002*	−0.002*
Book_to_Market	(−1.344)	(−1.382)	(−1.731)	(−1.778)
	−0.003	−0.003	−0.005	−0.006
Age	(−0.495)	(−0.537)	(−1.128)	(−1.161)
	0.000	0.000	0.000	0.000
R&D	(0.591)	(0.546)	(0.454)	(0.437)
			0.034**	0.033**
Leverage			(2.554)	(2.453)
			−0.059*	−0.059*
Past_Return			(−1.793)	(−1.819)
			0.000	0.000
Constant			(0.018)	(0.019)
	(−0.431)	(−0.583)	(−1.079)	(−1.106)
Observations	94	94	94	94
R-squared	0.029	0.037	0.155	0.158

Overall, the results do not provide strong support for Hypothesis 2b, as neither measure of CEO narcissism demonstrates a significant relationship with CARs. This suggests that investor anger is not directed at the CEO’s subjective attitudes or personality traits but is instead more focused on executive compensation levels and firm profits. The findings highlight the tension between public welfare and the pursuit of shareholder value maximization, which appears to be a central driver of negative market reactions.

### 4.7. Impact on the targeted industry

In this section, we test Hypothesis 3, which states that the stock market reaction to the assassination of UnitedHealth Group’s CEO was more severe for insurance firms in the same industry segment. These firms are classified under SIC code 6324, which corresponds to “Hospital & Medical Service Plans.” If investors were particularly concerned about firms with similar business models and practices to UnitedHealth, such as Elevance Health, Cigna Group, Humana Inc., and Centene Corp, then companies in the same sub-industry should show more negative cumulative abnormal returns (CARs). To test this hypothesis, first we compare the CARs between SIC 6324 (treated group) with insurance firms outside this sub-industry. We further conduct a matching analysis comparing firms in SIC 6324 (treated group) with insurance firms outside this sub-industry but similar in size and book-to-market ratio. Following the approach of Fu et al. [84], we select control firms that fall within 50% to 150% of each treated firm’s size and book-to-market ratio. From these, we identify the two closest matches using minimum Euclidean distance. For one treated firm, we could not find a control firm as per the matching criteria stated above. Thus, the process yields a matched sample of 9 treated firms and 18 control firms.

Table 8 reports the results. Panel A shows unadjusted comparisons between the treated and control groups. Firms in SIC 6324 experience significantly more negative cumulative abnormal returns (CARs) in the short-term event windows. The difference in CAR (0, + 2) is −2.764% and in CAR (1, +  2) is −2.977%, both statistically significant. Panel B presents results after matching. The short-term effects remain significant. The matched treated firms have a CAR (0, + 2) of −5.529%, compared to −2.222% for the control firms, a difference of −3.308% (significant at the 5% level). For CAR (1, +  2), the difference is −3.147%, significant at the 1% level. In contrast, the differences for longer event windows such as CAR (0, + 5), CAR (0, + 10), CAR (1, +  5), and CAR (1, +  10) are not statistically significant. It appears that the differentiating stock market reactions between SIC 6324 sub-group firms and other insurance firms are primarily observed with a very short window.

**Table 8:** Relationship between Insurance Segment and Cumulative Abnormal Returns (CARs) Following the Fatal Shooting of the CEO. This table presents the relationship between Insurance Segment and the cumulative abnormal returns (CARs) after the day of the shooting of the CEO of UnitedHealthcare. Panel A reports univariate differences in CARs between treated firms, defined as those in the ‘Hospital & Medical Service Plans’ insurance segment (SIC 6324), and control firms, which belong to other insurance industries. Panel B presents univariate comparisons where control firms are matched to treated firms based on firm size and book-to-market ratio. Panel C shows multivariate regression results where CARs are regressed on the dummy variable Hospital&Medical_Insurance, which equals 1 for firms classified under SIC 6324 and 0 otherwise. UnitedHealthcare and its competitors, including Elevance Health, Cigna Group, Humana Inc., and Centene Corp., are included in this category. The regression controls for firm characteristics such as size, book-to-market ratio, and firm age. Definitions of variables are provided in Appendix 1 (supplementary information file: S2 Appendix). Robust t-statistics are in parentheses. Asterisks *, ** and *** indicate the significance levels of 10%, 5% and 1%, respectively.

Panel A: Sample Before Matching						
CARs	Treated Firms		Control Firms		Difference	T-statistics
	Observations	Mean	Observations	Mean		
CAR (0 to +2)	10	−4.216%	124	−1.452%	−2.764%**	−2.419
CAR (0 to +5)	10	−6.875%	124	−3.534%	−3.342%	−1.308
CAR (0 to +10)	10	−11.158%	124	−6.988%	−4.170%	−1.121
CAR (1 to +2)	10	−3.928%	124	−0.951%	−2.977%***	−2.972
CAR (1 to +5)	10	−6.587%	124	−3.033%	−3.554%	−1.427
CAR (1 to +10)	10	−10.870%	124	−6.487%	−4.382%	−1.198
**Panel B: Sample After Matching**						
CARs	Treated Firms		Control Firms		Difference	T-statistics
	Observations	Mean	Observations	Mean		
CAR (0 to +2)	9	−5.529%	18	−2.222%	−3.308%**	−2.235
CAR (0 to +5)	9	−7.305%	18	−5.772%	−1.533%	−0.719
CAR (0 to +10)	9	−10.060%	18	−8.658%	−1.402%	−0.565
CAR (1 to +2)	9	−5.074%	18	−1.927%	−3.147%***	−3.350
CAR (1 to +5)	9	−6.850%	18	−5.477%	−1.373%	−0.875
CAR (1 to +10)	9	−9.605%	18	−8.364%	−1.242%	−0.623
**Panel C: Multivariate Regressions**						
	(1)	(2)	(3)	(4)		
VARIABLES	CAR(0,2)	CAR(0,2)	CAR(1,2)	CAR(1,2)		
Hospital&Medical_Insurance	−0.036***	−0.032**	−0.036***	−0.031**		
	**(−3.096)**	**(−2.477)**	**(−3.054)**	**(−2.352)**		
Size	−0.001	−0.001	0.001	0.001		
	(−0.575)	(−0.745)	(0.342)	(0.464)		
Book_to_Market	−0.008	−0.009	−0.006	−0.006		
	(−1.448)	(−1.608)	(−1.108)	(−0.980)		
Age	−0.000	−0.000	−0.000	−0.000		
	(−0.161)	(−0.286)	(−0.801)	(−0.726)		
R&D		0.006		0.015		
		(0.388)		(0.907)		
Leverage		−0.036		0.006		
		(−1.220)		(0.183)		
Past_Return		−0.001		0.004		
		(−0.211)		(0.866)		
Constant	0.002	0.006	−0.006	−0.028		
	(0.122)	(0.244)	(−0.455)	(−1.191)		
Observations	116	116	116	116		
R-squared	0.110	0.128	0.116	0.139		

Panel C of Table 8 shows multivariate regressions using the full sample of insurance firms. We regress short-term CARs on an indicator for SIC 6324 membership and control for firm size, book-to-market ratio, age, R&D intensity, leverage, and past stock returns. The SIC 6324 indicator remains negative and statistically significant for CAR (0, + 2) and CAR (1, +  2) windows. However, for other CAR windows, we do not observe any significant difference. These results indicate that firms in this segment experienced larger stock price declines during very short-term windows, even after adjusting for observable firm characteristics.

Overall, the evidence supports Hypothesis 3. The assassination triggered a sharper market response for firms in the Hospital & Medical Service Plans segment. This suggests that investors viewed these firms as sharing key similarities with UnitedHealth and expected them to face similar risks or public scrutiny.

### 4.8. Extending Reddit sentiment analysis to explain cross-firm variation in CARs

To further investigate whether firms associated with more negative social media sentiment experienced greater stock price declines, we construct firm-level measures of negativity exposure based on the Reddit discussions in Section 3. Specifically, we identify negative comments posted during the event window (0, + 2) that reference firms in our sample and count how many times each firm appears in comments. This procedure would yield a firm-level variable that captures the extent of public negativity exposure on social media, which allows us to test whether firms more frequently criticized in online discussions experienced larger stock price declines.

While this integration is conceptually intuitive, Reddit comments do not contain structured tags indicating which firm they refer to, and a direct name-based matching between comment text and company names can lead to mismatching. For instance, the word “citizen” in the comments may refer to U.S. citizens rather than the firm Citizen Inc. To address this challenge, we apply Named Entity Recognition (NER), a Natural Language Processing (NLP) technique that automatically detects and classifies entities mentioned in the text [85]. We then match the recognized organization entities to firm names in our sample based on exact string matches. This approach allows us to retain only those instances where a firm is explicitly identified as an organization, avoiding mismatches with common nouns that are spelled the same as company names [86]. While this approach limits the number of comments that can be linked to firms, it enhances the accuracy of our sentiment exposure variable.

Based on the mapping, we construct two measures of firm-level exposure to negative sentiment: [1] Negative_comments, which counts the number of negative Reddit comments in which a firm is mentioned, and [2] Negative_comments_dummy, an indicator equal to one if the firm is mentioned in at least one negative comment. We then regress the CARs on these measures to examine whether firms that are more frequently criticized on Reddit experience larger stock price declines. Table 9 reports the results. The coefficient on Negative_comments is negative and statistically significant at the 1% level in column [1] (–0.004), while Negative_comments_dummy yields a significant negative coefficient at the 1% level in column [2] (–0.037). The results remain robust after including additional controls, with significance levels at 10% and 5% in columns [3] and [4], respectively.

**Table 9:** Relationship between Negative Reddit Sentiment and Cumulative Abnormal Returns (CARs) Following the Assassination of the UnitedHealth CEO. This table presents the relationship between firm-level exposure to negative Reddit sentiment and cumulative abnormal returns (CARs) following the assassination of the CEO of UnitedHealthcare. The dependent variable is the cumulative abnormal return from day 0 to day 2. In columns (1) and (3), CAR is regressed on Negative_comments, which represents the number of negative Reddit comments mentioning the firm. In columns (2) and (4), CAR is regressed on Negative_comments_dummy, an indicator equal to one if the firm is mentioned in at least one negative Reddit comment, and zero otherwise. Several firm accounting and stock characteristics are controlled for in the regressions, including firm size, book-to-market ratio, and firm age. Definitions of all variables are provided in Appendix 1 (supplementary information file: S2 Appendix). Robust t-statistics are reported in parentheses. Asterisks *, **, and *** denote significance at the 10%, 5%, and 1% levels, respectively.

	(1)	(2)	(3)	(4)
Variables	CAR	CAR	CAR	CAR
Negative_comments	−0.004***		−0.004*	
	**(−2.889)**		**(−1.980)**	
Negative_comments_dummy		−0.037***		−0.031**
		**(−3.337)**		**(−2.561)**
Size	−0.001	−0.001	−0.001	−0.001
	(−0.478)	(−0.383)	(−0.665)	(−0.628)
Book_to_Market	−0.006	−0.006	−0.007	−0.007
	(−1.162)	(−1.218)	(−1.409)	(−1.412)
Age	0.000	0.000	0.000	0.000
	(0.318)	(0.208)	(0.192)	(0.042)
R&D			−0.004	0.005
			(−0.222)	(0.325)
Leverage			−0.041	−0.037
			(−1.358)	(−1.254)
Past_Return			−0.000	−0.001
			(−0.062)	(−0.211)
Constant	−0.005	−0.005	0.010	0.001
	(−0.325)	(−0.340)	(0.385)	(0.048)
Observations	116	116	116	116
R-squared	0.082	0.083	0.101	0.102

These findings suggest that investors reacted more severely to firms that were specifically named and criticized in online Reddit discussions. In line with our interpretation, social media narratives appear to channel public outrage in a targeted manner, amplifying stock price declines for firms perceived to be more blameworthy.

### 4.9. Placebo test using sudden CEO deaths in other industries

To further validate our interpretation of the assassination’s industry-wide impact through the lens of social bandit theory, we examine whether the sudden death of CEOs in other industries triggered similar market responses among peer firms. Specifically, we test the market reaction within the same industry following two high-profile CEO death events. These events also triggered media coverage and public attention but did not generate widespread societal backlash or industry-level moral criticism. This contrast helps highlight the distinctive nature of the health insurance case and underscores the role of public outrage and investor sentiment in shaping abnormal stock returns.

Panel A of Table 10 analyzes the market reasons in the transportation industry following the fatal shooting of Philip Trenary, former CEO of Pinnacle Airlines and a prominent civic figure in Memphis. Although the incident attracted media coverage due to Trenary’s community profile, it did not spark strong public discussion about the industry’s conduct or moral concerns. Event study results show a small and marginally significant positive CAR of 0.76% over the (0, + 2) window (significant at 10% level), with no significant effects in other windows.

**Table 10:** Sudden CEO Deaths in Other Industries – Univariate CAR Analysis. This table reports the cumulative abnormal returns (CARs) for industry peer firms surrounding the sudden deaths of CEOs in two different industries. Panel A presents CARs for transportation firms around the fatal shooting of Philip Trenary, the former CEO of Pinnacle Airlines, on September 27, 2018. Panel B presents CARs for electronic equipment firms around the death of Micron Technology’s active CEO, Steve Appleton, who died in a plane crash on February 3, 2012. Peer firms are defined by the same Fama-French 48 industry classification as the firm experiencing the CEO death. ***, **, and * indicate statistical significance at the 1%, 5%, and 10% levels, respectively.

Panel A. Market Reaction in the Transportation Industry to the Shooting of the Former Pinnacle Airlines CEO				
	# observations	Mean (%)	t-stat	p-value
CAR (0 to +2)	165	0.755%	1.925*	0.054
CAR (0 to +5)	165	−0.273%	−0.629	0.530
CAR (0 to +10)	165	0.319%	0.611	0.541
CAR (1 to +2)	165	−0.953%	−1.129	0.259
CAR (1 to +5)	165	0.519%	1.530	0.126
CAR (1 to +10)	165	0.083%	0.170	0.865
**Panel B. Market Reaction in the Electronic Equipment Industry to the Death of Micron Technology’s CEO**				
	# observations	Mean (%)	t-stat	p-value
CAR (0 to +2)	274	0.424%	1.170	0.242
CAR (0 to +5)	274	1.398%	3.064***	0.002
CAR (0 to +10)	274	1.796%	3.255***	0.001
CAR (1 to +2)	274	1.727%	2.470**	0.014
CAR (1 to +5)	274	0.077%	0.253	0.800
CAR (1 to +10)	274	1.450%	2.816***	0.005

Panel B investigates the electronic equipment industry in response to the accidental death of Steve Appleton, then-CEO of Micron Technology, in a 2012 plane crash. As an active CEO, Appleton’s death was widely reported. However, public reaction remained focused on the personal tragedy rather than corporate behavior. CARs for peer firms were positive and statistically significant in a variety of windows: 1.40% over the (0, + 5) window (significant at 1% level), and 1.80% over (0, + 10) (significant at 1% level).

These findings are not intended to suggest that the CEO deaths in other industries necessarily produce large industry-wide stock price effects. Rather, the absence of significant negative CARs in these cases reinforces our central argument. Both events lacked the strong public outrage, moral condemnation, and narrative spillover that are central to the social banditry mechanism. Without a wave of collective indignation directed at the industry, investor sentiment remained relatively unaffected. Unlike other high-profile CEO deaths, the assassination of the UnitedHealth CEO sparked rare collective outrage toward the industry, fueling investor pessimism and driving the significant negative spillovers we document.

### 4.10. Robustness tests using extended event windows

We examine the robustness of our findings by re-estimating the main regressions using extended event windows of (0, + 3) and (0, + 5). Across specifications in the untabulated results, the coefficients on high revenue growth, media attention, and compensation-related variables remain statistically significant and directionally consistent with those in the baseline (0, + 2) window. While the coefficients on CEO narcissism remain insignificant. Notably, for these significant variables, the magnitudes of the coefficients decrease in the (0, + 3) window but increase again in the (0, + 5) window, ultimately exceeding their baseline levels. This non-monotonic pattern aligns with the cumulative abnormal return trajectory in Fig 2, which shows a temporary rebound in the market around day 3 before continuing its downward trend.

In contrast, the coefficient on profitability remains negative but becomes statistically insignificant in the extended windows. A likely explanation is that high-profitability firms are perceived as fundamentally undervalued after initial price declines and are more likely to attract speculative contrarian traders, thereby offsetting the early sell-off [87,88]. As a result, the early negative effect of profitability becomes muted over the longer window due to countervailing upward price pressure. Moreover, expanding the event window increases the risk that unrelated news adds noise to abnormal return estimates [11]. Still, the coefficient on profitability continues to decline and becomes more negative in the (0, + 5) window compared with (0, + 3), suggesting that the market was torn between selling on negative sentiment pressure and buying on high profit fundamentals during the rebound, rather than ignoring profitability altogether in the longer windows.

## 5. Discussion and conclusion

Maximizing shareholder value has long been considered the fundamental goal of corporate management, a principle rooted in over 200 years of research in economics and finance [42]. Furthermore, as Jensen (42, p. 302) argues, “social welfare is maximized when all firms in an economy maximize total firm value.” If this principle holds true, the management team of UnitedHealthcare could be seen as having performed their roles effectively, as the company achieved substantial profitability and growth under their leadership. However, the shooting incident on December 4th cast significant doubt on this seemingly impeccable performance, highlighting the deeper societal frustrations with profit-driven corporate practices, particularly in the insurance industry. According to the ‘social banditry theory’, the prevalence of inequitable systems can unintentionally become a symbol of a community’s frustration, which may manifest through public backlash and trigger negative investor sentiment, ultimately contributing to a stock market debacle. In this paper, we aim to empirically examine these tensions by undertaking two strands of analysis to empirically investigate the factors contributing to the decline in stock returns among publicly listed U.S. insurance firms following the assassination of UnitedHealthcare’s CEO.

We first investigate public viewpoints on the incident using topic modeling based on 59,644 Reddit comments, identifying nine key topics that encapsulate the narratives surrounding the event. These topics highlight public anger toward the health insurance industry and its profit-driven focus, support for the perpetrator alongside criticism of the CEO, frustration over patient bills, and broader debates on Medicaid and the healthcare system. Furthermore, our sentiment analysis reveals that public discussions surrounding the event are predominantly negative, reflecting widespread dissatisfaction with the profit-driven practices of the insurance industry. Such frustration is further reflected in the stock market’s response, as our CAR analysis shows significant negative returns for insurance companies across various time windows following December 4th.

We then conduct a series of empirical analyses aligned with our hypotheses to directly examine the factors driving the decline in stock prices among insurance companies. The results reveal that perceived corporate profitability, as proxied by profit and revenue growth, and firm glamour, characterized by the media attention associated with news coverage, contribute significantly to negative abnormal returns. Furthermore, high compensation levels for CEOs and other executives are linked to a more pronounced negative impact on abnormal stock returns. However, we did not find evidence linking CEO narcissism to the negative stock impact. This finding suggests that public concern is not directed at the CEO’s personal attitudes but rather at the underlying business model and profit-driven practices of the company. Additionally, the results show that insurance companies in the ‘Hospital & Medical Service Plans’ sector, which are most closely associated with providing essential healthcare services and directly impacting individuals, experienced the most significant declines in stock returns. This further reinforces the notion that systemic issues within this industry segment, particularly those tied to its profit-driven practices, are at the core of public dissatisfaction and market reactions.

Our paper contributes to the ongoing debate between shareholder value maximization and stakeholder theory by examining the potential consequences of prioritizing profit over stakeholder interests, particularly within the insurance industry. It explores how customers, as a vital group of stakeholders, may be overlooked and negatively impacted under the current profit-driven business model [38]. The findings offer valuable insights for managers, regulators, shareholders, and policymakers by identifying key issues that resonate with the public and highlighting the firm and executive characteristics that generate the greatest concern. Yet, we acknowledge that if the negative economic impact of this shooting incident does not sustain for a long period, it may not motivate the managers, regulators, and policymakers to take significant action. Overall, by integrating ‘social banditry theory’ and investor sentiment into the financial context, our study emphasizes the importance of understanding how societal frustration can translate into market consequences. Focusing on the insurance sector, our study emphasizes the need for more balanced and sustainable corporate strategies that address both shareholder value and stakeholder well-being in industries directly tied to public welfare.

Our study also contributes to the literature on market reactions to sudden CEO deaths. Prior studies have used such events to estimate a CEO’s influence on firm value or to evaluate corporate governance structures. For instance, using a sample of 240 CEO deaths, one study finds that investors have attributed increasing importance to individual CEOs in recent years, with market reactions becoming more pronounced over time [10]. Another line of work shows that stock price responses vary systematically with the degree of managerial entrenchment, suggesting that governance quality shapes how markets interpret CEO deaths [11]. Our study complements this literature by analyzing an assassination, a rare instance of CEO turnover, and differs in the explanatory framework. Rather than using executive deaths as a natural experiment to infer the individual CEO’s value to the firm [10–13,89], we focus on how public outrage and investor sentiment shape market reactions beyond firm-level fundamentals. In doing so, we also identify the firm and CEO characteristics that amplify these effects, offering new insights into the reputational channels through which socially resonant events impact financial markets.

While our study focuses on short-term market reactions driven by investor sentiment, it is important to recognize that stock prices eventually reflect fundamental performance [36]. For example, UnitedHealth’s stock rebounded in early April 2025 after the government announced higher-than-expected Medicare Advantage reimbursement rates for 2026, signaling renewed confidence in future earnings [90]. In contrast, in mid-April 2025, the company’s stock experienced its steepest single-day drop in decades following a disappointing earnings report that revealed rising medical costs and operational disruptions [91]. These cases show that while investor sentiment can create temporary volatility, long-term valuation depends on underlying financial fundamentals. To translate public frustration into lasting improvements in both stakeholder and shareholder outcomes, public sentiment must ultimately be channeled into concrete regulatory reforms that reward corporate behaviors attentive to stakeholder interests and long-term social welfare.

While our study provides valuable insights, it is not without limitations. First, our topic modeling relies exclusively on comments collected from the Reddit platform. This data source is limited in scope due to platform-specific restrictions and sample size constraints. Future research could address this limitation by incorporating textual data from other social media platforms and traditional news outlets. Second, our investigation focuses primarily on firm characteristics related to perceived corporate profitability and glamour and CEO traits concerning compensation and narcissism. However, other significant characteristics, such as board composition, corporate social responsibility efforts, and governance practices, remain unexplored. Future research could expand the scope to include these factors for a more comprehensive understanding of market reactions.

## Supporting information

S1 Data This supplementary information file (S1 Data (Excel file)) presents data to replicate the descriptive and regression results in the paper. These are the key findings of the paper. Since we have used commercial databases (e.g., COMPUSTAT, CRSP) to retrieve firms’ financial information, we have removed firm identities from these worksheets.
(XLS)

S2 Appendix This supplementary information file (S2 Appendix (Word file)) contains variable descriptions for variables used in the analyses. (DOCX)
