# Supplementary material for: Delay, deny, and defend: Public outrage at health insurance companies and stock market debacle
Source: PLoS One. 2025 Oct 14;20(10):e0334399. doi: 10.1371/journal.pone.0334399 (PMC12520363; doi:10.1371/journal.pone.0334399)
Supplement: S2 Appendix — (DOCX) [file pone.0334399.s002.docx]

**Appendix 1: Variable Description**

| Variable Name | Description |
| --- | --- |
| CAR | The Cumulative Abnormal Return (CAR) measures the sum of abnormal returns over a three-day window, capturing the deviation of a stock's return from its expected performance based on the market model. |
| Profit | Calculated as the ratio of income before extraordinary items to total assets. |
| High_Rev_Growth | High revenue growth indicator, a binary indicator that equals 1 if the firm’s average revenue growth rate over the past three years exceeds the sample median, and 0 otherwise. |
| Media_1year | Median attention, measured as the total number of news articles (in thousands) mentioning the firm in the past one year in the Factiva database. |
| Media_3year | Median attention, measured as the total number of news articles (in thousands) mentioning the firm in the past three years in the Factiva database. |
| CEO_Compensation | The logarithm of a CEO’s total compensation. |
| Excu_Avg_Compensation | The logarithm of average compensation for all executives reported in the firm's filings. |
| Excu_Total_Compensation | The logarithm of total compensation for all executives reported in the firm's filings. |
| CEO_Narcissism | CEO narcissism score is calculated as the natural logarithm of one plus the ratio of first-person singular pronouns to all first-person pronouns used by the CEO during the Q&A section of earnings calls. The annualized CEO’s narcissism is the average narcissism score over the recent four quarters. |
| First_Pronoun | The percentage ratio of first-person singular pronouns used by the CEO during the Q&A section of earnings calls. |
| Hospital&Medical_Insurance | A dummy variable that equals 1 if the firm belongs to the same industry segment as UnitedHealthcare, identified by SIC code 6324. |
| Size | The size of the firm, defined as the natural logarithm of market value of equity. |
| Book_to_Market | Ratio of the book value of equity to market value of equity. |
| Age | The age of the firm, calculated as the difference between current year and the firm's first appearance in CRSP. |
| R&D | A binary variable that takes a value of 1 if a firm has positive research and development expenditure and 0 otherwise. |
| Leverage | Ratio of long-term debt to total assets. |
| Past_Return | The buy-and-hold stock returns in the past one year. |
| Negative_comments | Number of negative Reddit comments that mention the firm during the initial three-day event window. |
| Negative_comments_dummy | Indicator equal to one if the firm is mentioned in at least one negative Reddit comment during the initial three-day event window, and zero otherwise. |
| We lag our explanatory variables by one year in our main regressions. | |
|  |  |
